# Regulatory Roles of Noncoding RNAs in the Progression of Gastrointestinal Cancers and Health Disparities

**DOI:** 10.3390/cells11152448

**Published:** 2022-08-07

**Authors:** Aditi Kulkarni, Sharan Gayathrinathan, Soumya Nair, Anamika Basu, Taslim A. Al-Hilal, Sourav Roy

**Affiliations:** 1 Department of Biological Sciences, University of Texas at El Paso, El Paso, TX 79968, USA; 2 Border Biomedical Research Center, University of Texas at El Paso, El Paso, TX 79968, USA; 3 Copper Mountain College, Joshua Tree, CA 92252, USA; 4 Center for Health Disparities and Molecular Medicine, Loma Linda University School of Medicine, Loma Linda, CA 92350, USA; 5 Department of Pharmaceutical Sciences, School of Pharmacy, University of Texas at El Paso, El Paso, TX 79968, USA

**Keywords:** gastrointestinal cancers, noncoding RNAs, chemoresistance, biomarkers, therapeutic targets, disparities

## Abstract

Annually, more than a million individuals are diagnosed with gastrointestinal (GI) cancers worldwide. With the advancements in radio- and chemotherapy and surgery, the survival rates for GI cancer patients have improved in recent years. However, the prognosis for advanced-stage GI cancers remains poor. Site-specific GI cancers share a few common risk factors; however, they are largely distinct in their etiologies and descriptive epidemiologic profiles. A large number of mutations or copy number changes associated with carcinogenesis are commonly found in noncoding DNA regions, which transcribe several noncoding RNAs (ncRNAs) that are implicated to regulate cancer initiation, metastasis, and drug resistance. In this review, we summarize the regulatory functions of ncRNAs in GI cancer development, progression, chemoresistance, and health disparities. We also highlight the potential roles of ncRNAs as therapeutic targets and biomarkers, mainly focusing on their ethnicity-/race-specific prognostic value, and discuss the prospects of genome-wide association studies (GWAS) to investigate the contribution of ncRNAs in GI tumorigenesis.

## 1. Introduction

Gastrointestinal (GI) cancers account for more than 26% (4.8 million new cases) of the incidence rates and approximately 35% (3.4 million deaths) of all cancer-related deaths worldwide [1]. These numbers are predicted to increase by more than 50%, respectively, by 2040 [2]. Cancers of the stomach (approximately 1.0 million new cases), liver (840,000 cases), esophagus (570,000 cases), pancreas (460,000 cases), and colorectum (1.8 million cases) form the principal malignant conditions of the GI tract, which costs billions of dollars for treatment annually [3] and are imposing major challenges to public health [4]. Though the survival rates have improved with the advancements in radio- and chemotherapy and surgery, the prognosis for advanced-stage GI cancers remains poor. Therefore, new screening methods and therapeutic targets are required to improve GI cancer patient survival. With the advancement in cancer genomics, it is evident that most of the mutations or copy number changes associated with carcinogenesis are largely found in noncoding DNA regions [5,6]. Originally referred to as the “junk DNA”, they are now known to transcribe several noncoding RNAs (ncRNAs), which are implicated to regulate cancer initiation, metastasis, and drug resistance [7,8,9]. Some ncRNAs, such as lncRNA H19, miR-29a, miR-29b, and miR-29c, act as oncogenes or tumor suppressors [10]; however, the exact function and mechanism of action for most ncRNAs are still unknown. Based on the structure, ncRNAs are classified further to include microRNAs (miRNAs), small interfering RNAs (siRNAs), antisense RNAs (asRNAs), long noncoding RNAs (lncRNAs), and circular RNAs (circRNAs) [11]. In this review, we summarize the regulatory functions of ncRNAs in GI cancer development and progression, chemoresistance, and health disparities. Additionally, we highlight their potential as therapeutic targets or biomarkers and discuss the prospects of genome-wide association studies (GWAS) to investigate the contribution of ncRNAs in GI tumorigenesis.

## 2. ncRNA-Mediated Regulation of Cell Signaling Pathways in GI Cancer Progression

**Figure 1:**
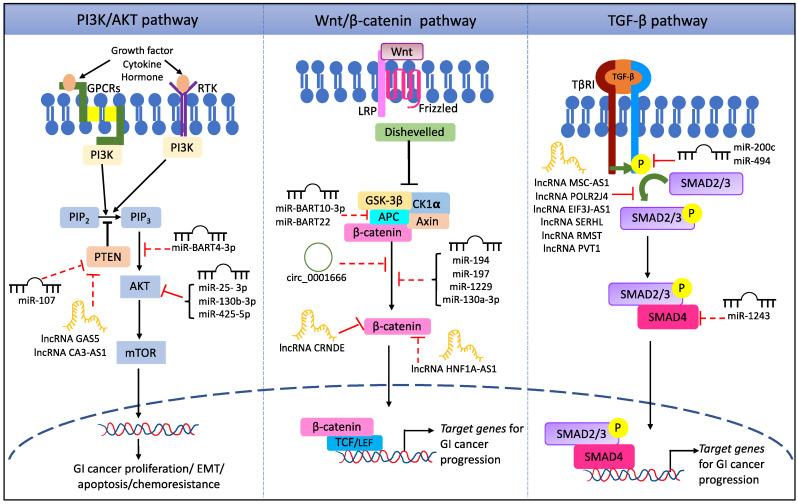
NcRNA-mediated regulation of signal transduction pathways in GI cancer progression. Representative illustration of different ncRNA-mediated dysregulation of A. PI3K/AKT, B. Wnt/β-catenin, and C. TGF-β pathways. It should be noted that the ncRNA-mediated dysregulation of signal transduction pathways is not limited to the above pathways or ncRNAs; the red dotted line represents inhibition, and the red continuous frontal line represents activation by the respective ncRNA(s).

During cancer development and progression, ncRNAs have been reported to have key regulatory functions. They are known to dysregulate several signaling pathways to promote cell proliferation, differentiation, and epithelial–mesenchymal transition (EMT) in various cancer types. LncRNAs and miRNAs are the most extensively studied ncRNAs in cancer research [12]. The lncRNA is a RNA molecule with transcript length of more than 200 nt [13], whereas miRNAs are smaller in length, usually around 18–25 nt [14]. In addition, miRNAs interact with lncRNAs and many downstream target genes to control their expression. LncRNAs act as “sponges” and sequester the miRNAs, eventually silencing them and their regulatory cascades [15]. In this section, we focus on the commonly sighted pathways, namely phosphoinositide 3-kinase/protein kinase B (PI3K/AKT), Wnt/β-catenin, transforming growth factor-β (TGF-β), nuclear factor kappa B (NF-κB), Notch, Hippo, and Ras/Raf/Mitogen-activated protein kinase/extracellular-signal-regulated kinase (Raf/MEK/ERK), that are dysregulated by several ncRNAs to facilitate the progression of various GI cancers (Figure 1).

### 2.1. ncRNAs Regulate the PI3K/AKT Signaling Pathway

The PI3K/AKT signaling pathway plays a crucial role in the progression of GI cancers through various biological processes such as proliferation, metastasis, chemo- and radioresistance, or autophagy. In general, ncRNAs can regulate the PI3K/AKT signaling by directly targeting AKT in a few cases, while most of them target the negative regulators of this pathway. For example, in pancreatic ductal adenocarcinoma (PDAC), when upregulated in PDAC tissues and cells, miR-107 induces cell migration, invasion, and EMT by downregulating tumor suppressors *caveolin-1* and *PTEN* genes and by upregulating p-AKT [16]. However, certain miRNAs such as miR-34a and miR-125a-5p act as tumor suppressor miRNAs and induce apoptosis and reduce hepatocellular carcinoma (HCC) proliferation and metastasis [17]. Similarly, in Epstein–Barr-virus-associated gastric cancer (EBVaGC), miR-BART4-3p targets *AXL*-mediated PI3K/AKT activation to inhibit EMT in gastric cancer cells [18]. This pathway is also known to be associated with the M2 polarization of macrophages. Tumor-activated macrophages (TAMs) that are typically maintained in the more polarized M2 state have anti-inflammatory and tumor-promoting functions and are associated with pro-metastatic cancer phenotype [19]. Several miRNAs, such as miR-25-3p, miR-130b-3p, and miR-425-5p, are transferred by exosomes to colorectal cancer (CRC) cells via the CXCL12/CXCR4 axis to activate the PI3K/AKT signaling pathway to induce the M2 polarization of macrophages, which increases EMT and vascular endothelial growth factor (VEGF) secretion in CRC cells [20]. Additionally, lncRNA H19 regulates the PI3K/AKT pathway by functioning as a competing endogenous RNA and predicts poor prognosis in CRC patients [21]. This pathway is also known to be modulated by circular ncRNAs, such as circ_NRIP1, which increases colony formation, cell viability, migration, and invasion in esophageal cancer [22]. With the advancement in microarray and next-generation sequencing technologies, tools are available to display PI3K/AKT-reprogrammed ncRNA profiles. It would be interesting to integrate the ncRNA profiling and ncRNA target prediction tools to establish the link between PI3K, AKT, and ncRNAs to comprehensively understand the PI3K/AKT gene regulatory mechanisms, thereby exploring GI cancer pathogenesis and diagnosis in a personalized manner, as well as developing new therapeutic strategies.

### 2.2. ncRNAs Regulate the Wnt/β-Catenin Signaling Pathway

The Wnt/β-catenin signaling pathway is another pathway that is highly modulated by ncRNAs to regulate cellular proliferation, differentiation, migration, genetic stability, and apoptosis in GI cancers. The aberrant activation of the Wnt pathway dysregulates the multifunctional protein β-catenin in GI cancers [23,24]. In addition, miRNA-20b and lncRNA NNT-AS1 are known to be upregulated in gastric cancer cells to promote cell proliferation, migration, and invasion via the Wnt signaling pathway [25,26]. In EBVaGC, miRNAs miR-BART10-3p and miR-BART22 play important roles in metastasis by activating the Wnt signaling pathway and targeting the adenomatous polyposis coli (*APC)* and Dickkopf-related protein 1 (*DKK1*) genes [27]. The Wnt/β-Catenin signaling pathway is also dysregulated in hepatocellular carcinoma cells and tissues. The miRNAs miR-19a-3p/miR-376c-3p suppress the target gene *SOX6* to activate the Wnt/β-catenin pathway [28]. These observations indicate that the differential regulation of the Wnt/β-catenin pathway by specific ncRNAs plays an important role in GI cancer biology and could act as oncogenes. However, miR-194, miR-197, circ_0001666, miR-1229, and miR-130a-3p have been shown to inhibit the Wnt signaling pathway, preventing the progression of various GI cancers [29,30,31,32]. This trend of the up- and downregulation of the Wnt/β-catenin pathway by specific ncRNAs is observed in GI cancers; however, further studies are required to understand the underlying mechanisms to promote an efficient therapeutic strategy by targeting ncRNAs associated with the Wnt pathway in GI cancers.

### 2.3. ncRNAs and the Other Signaling Pathways

Other pathways, including TGF-β, Hippo, MAPK, NF-κB, Hedgehog, mTOR, and Raf/MEK/ERK pathways, are also found to be dysregulated by ncRNA during GI cancer progression; however, the number of studies is limited. The TGF-β signaling pathway is an evolutionarily conserved pathway that controls cell growth, differentiation, and development in various biological systems. In cancer cells, TGF-β performs a dual role via SMAD to either promote tumor suppression or inactivate the immune system to promote tumorigenesis that leads to changes in cell differentiation, causing epithelial–mesenchymal transition (EMT) [33]. A few miRNAs (*n* = 17) are known to be upregulated in hepatocellular carcinoma, of which miR-494 targets the SIRT3 and TGF-β/SMAD signaling pathways to promote cell proliferation and migration of hepatoma cells [34]. Similarly, miR-200c also targets the TGF-β1/zinc-finger E-box-binding homeobox (ZEB1) pathway to induce EMT and promote cellular dissemination from the primary tumor and subsequent metastasis in CRC [35].

The dysregulation of the Hippo pathway, which controls cell growth, proliferation, and apoptosis, is associated with cancer development. In CRC, tumor suppressor miRNAs miR-30a-5p [36] and miR-375-3p [37] have been associated with the downregulation of the Hippo signaling pathway, limiting CRC proliferation, invasion, and migration. In the case of miR-375, it downregulates the yes1-associated transcriptional regulator (*YAP1)* gene, resulting in a reduced expression of connective tissue growth factor (*CTGF*), *cyclin D1,* and baculoviral inhibitor of apoptosis-repeat-containing 5 (*BIRC5*) target genes that are downstream of the Hippo–YAP1 pathway [37]. On the other hand, a circRNA hsa_circ_0128846 is seen to be upregulated in CRC tissues [38], which sponges miR-1184 to upregulate the Ajuba LIM protein (*AJUBA)* gene, which upregulates the Hippo–YAP1 pathway to promote CRC proliferation.

In pancreatic cancer, miR-143 acts as a tumor suppressor by targeting the transforming growth factor (TGF)-β-activated kinase 1 (TAK1) to inactivate the MAPK and NF-κB pathways, which subsequently prevents cell proliferation and migration and induces apoptosis and G1/S arrest [39]. A similar phenomenon is observed in liver cancer cells, where miR-129-5p inhibits the MAPK pathway by targeting the calcium calmodulin-dependent protein kinase IV (*CAMK4*) to reduce tumor progression [40]. The mammalian target of the rapamycin (mTOR) signaling pathway is downregulated by the tumor suppressor miR-195, which limits the proliferation of ECC cells by targeting the major nonhistone chromosomal protein that controls cell cycle, transformation, proliferation, and apoptosis, the high-mobility group protein A2 (*HMGA2*) gene [41]. On the contrary, miR-132 and lncRNA AL139002.1 upregulate the Hedgehog and MEK/ERK signaling pathways in pancreatic and gastric cancer, respectively [42,43]. Specifically, miR-132 targets the sonic hedgehog (*Shh*) gene to induce the proliferation of pancreatic cells by reducing the expressions of Caspase-3 and Caspase-9, thus suppressing cell apoptosis [42]. The hepatitis A virus cellular receptor 1 (*HAVCR1*) gene is targeted by lncRNA AL139002.1 in gastric cancer cells to activate cell proliferation via the MEK/ERK signaling pathway [43].

These studies clearly indicate the roles of ncRNAs in regulating several biological pathways that contribute to the fate of tumor development. With the rapid progress in ncRNA and RNA biopharmaceutical research, ncRNA-targeted therapies could be considered a promising alternative to surgical methods, especially for advanced GI cancers, for which treatment options are currently limited.

## 3. Role of ncRNAs in Chemoresistance in GI Cancers

Chemotherapy, a treatment approach predominantly practiced for the annihilation of cancerous cells by obstructing cellular growth and division, includes a cocktail of drugs such as adriamycin, platinum-based drugs, 5-fluorouracil (5FU), vincristine, and paclitaxel [44]. However, the development of chemoresistance remains a challenge for patients receiving chemotherapy, preventing better recovery rates for GI cancer patients. Cancer patients exhibit intrinsic or acquired chemoresistance by a multistep process leading to interference with the cellular function. Network analyses have revealed that mechanisms underlying the roles of ncRNA-mediated chemoresistance are highly complex. The abnormal expression of ncRNA promotes the manifestation of chemoresistance by inactivating apoptosis signaling pathways, hindering cell cycle checkpoints, increasing cell proliferation, autophagy, DNA damage repair, cancer stem cells (CSCs), and EMT [44]. The ncRNAs, mostly the miRNAs and lncRNAs, have a pivotal role in inducing chemoresistance in GI cancers. These two families of ncRNAs commonly tend to target the cell cycle and several different signaling pathways (MAPK/ERK, PI3K/AKT, Wnt/B-catenin, Hippo, NF-κB, and Notch) to confer drug(s) resistance in GI cancers. Table 1 lists the different ncRNA(s) that contribute to drug resistance in different GI cancers, along with their known molecular targets mediating the drug/multidrug resistance. Interestingly, lncRNAs such as lncRNA CRNDE, GAS5, and HOTAIR are seen to contribute to 5FU, adriamycin, oxaliplatin, cisplatin, gemcitabine, and/or doxorubicin resistance in multiple GI cancers by targeting different molecules/pathways (Table 1). For example, lncRNA CRNDE contributes to 5FU, oxaliplatin, and adriamycin resistance in CRC, gastric cancer, and HCC by targeting *β-catenin* and *TCF4, PICALM,* and *CELF2* and *LATS2*, respectively [24,45,46]. Conversely, multiple ncRNAs lncRNA CRNDE, lncRNA PCAT6, lncRNA SNHG6, miR-125b, miR -26a-5p, and miR -532-3p contribute to 5FU resistance in CRC by targeting *β-catenin* and *TCF4, HMGA2, ULK1, APC, ULK1, ETS1*, and *TGM2* genes, respectively [24,47,48,49]. From these studies, it is clear that ncRNAs do have an apparent impact on modulating CRC chemoresistance in GI cancers. However, the number of studies conducted is limited, and we still lack a clear understanding of the mechanisms that regulate ncRNA-based chemoresistance in different GI cancers. It is crucial to further identify different ncRNAs and their upstream or downstream mediators to overcome the limitations of ineffective chemotherapy, relapse, and mortality in GI cancer patients.

## 4. Genome-Wide Profiling of ncRNAs in GI Cancers

Genome-wide profiling of ncRNAs has garnered significant attention from researchers studying GI cancers due to their crucial role in transcriptional and post-transcriptional regulation. Novel miRNA-based signatures for the detection and prognosis of metastasis in GI cancers are established in many studies that show a promising future in understanding the development and treatment of patients. A genome-wide transcriptome profiling conducted by Shimura et al., in 2021, identified five miRNAs in the initial filtering phase, but only three miRNAs (miR-30a-5p, -659-3p, and -3917) were significantly overexpressed in primary tumors from peritoneal metastasis in gastric cancer patients, making them potential miRNA signatures to identify peritoneal metastasis in gastric cancer patients [73]. In CRC progression, Kalmár et al. (2019) checked for lncRNA expression levels in colonic cancer biopsy samples and compared those with controls, which were further analyzed with Human Transcriptome Array (HTA). Sixteen lncRNAs were differentially expressed, including LINC02023, MEG8, AC092834.1, which were downregulated, and CCAT1 and CASC19 were upregulated [74]. Similar studies have also revealed the group of lncRNAs that promote liver metastasis in CRC patients [75]. A study involving the transcriptomic profiling of HCC tissues, performed via high-throughput RNA sequencing, found 214 differentially expressed lncRNAs, of which the expression of 4 lncRNAs (NONHSAT003823, NONHSAT056213, NONHSAT015386, and especially NONHSAT122051) is correlated with tumor cell proliferation, portal vein tumor thrombosis, and serum or tissue alpha-fetoprotein levels [76]. Further, genome-wide transcriptomic screening could also be performed from databases such as The Cancer Genome Atlas (TCGA). A large number of studies have identified ncRNAs from the TCGA database that are associated with a poor prognosis of patients with esophageal adenocarcinoma (EADC) and esophageal squamous cell carcinoma (ESCC) [77] or are associated with tumor size, N classification, clinical stage, or the risk of esophageal cancer recurrence [78,79]. Interestingly, the silencing of lncRNA-KIAA1244-2 identified in this study in esophageal cancer cells is known to inhibit cell proliferation and *TNFAIP3* expression; thus, it could be a potent therapeutic target for ESCC [79]. The TCGA database has also been analyzed to identify the expression profiles of lncRNAs in HCC tissues, among which two crucial lncRNAs termed “PVT1” and “SNHG7” were found to be involved in the recurrence of the tumor, and lncRNA unigene56159 was found to promote the migration and invasion of HCC cells through the miR-140-5p/SNAI2 axis, where it acted as a competing endogenous RNA (ceRNA) for miR-140-5p and downregulated its expression [80]. Few genome-wide lncRNA-microarray studies have been performed with gastric cancer patients’ plasma and compared their results with healthy control plasma to identify lncRNA signatures, namely FAM49B-AS, GUSBP11, CTDHUT, TINCR, CCAT2, AOC4P, BANCR, and LINC00857, which are upregulated in the plasma from gastric cancer patients [81,82]. Implementing the screening of these lncRNAs from patient plasma for clinical diagnostic purposes would provide a promising noninvasive approach to detect gastric cancer in the future.

These studies conducted with different GI tumor samples have provided us with the dysregulated patterns of various lncRNAs; however, it is crucial to construct a ceRNA network to identify survival- and prognosis-related ncRNAs to understand GI cancer pathogenesis. In recent years, efforts have been made to develop ceRNA networks for correlating 77 lncRNAs [83], and 32 miRNAs with the overall survival and prognosis of HCC patients [84]. Despite the success of GWAS in the identification of hundreds of genetic ncRNAs associated with GI cancers, further comprehensive studies are required to provide more insights into the tumor progression and help develop screening tools for early GI cancer detection.

## 5. ncRNAs as Potential Therapeutic Targets or Biomarkers for GI Cancer Progression

In recent years, the study of the tumor microenvironment has played a crucial role in understanding the disease progression [85]. Current research shows a considerable amount of evidence of ncRNAs undergoing significant changes in GI cancer progression that can potentially be used as predictive biomarkers or prognostic measures. The ncRNAs open a wide array of scope for clinical advancement with diagnostic information retrieved with noninvasive, sensitive, and specific disease development detection. Advances in the prognostic prediction of gastrointestinal cancer using ncRNAs are rapidly progressing with recent advances, including using electrical biosensors as a promising alternative to developing fast and low-cost detection systems to quantify mRNA biomarkers [86]. Although new technologies and treatments are progressing toward increasing the survival rates and treatments, the diagnosis of various types of cancers in patients is performed only when the disease has progressed to advanced stages. The aberrant expression of ncRNAs is associated with various cancers, and their ability to regulate the expression of various downstream target genes and their associated pathways has provided a rationale to pursue them for their untapped potential in early-stage biomarker and therapeutic drug development in GI cancers [87]. Preclinical studies have demonstrated the potential antitumor activity of synthetic miRNA- or lncRNA-based therapeutic molecules, with some showing promising results even in early-phase human clinical trials [88,89]. However, as the studies using ncRNA-based cancer therapeutics continue to evolve, there is a lot to unravel and understand about the precise molecular mechanisms and specific downstream therapeutic targets. Here, we summarized the identified lncRNAs and miRNAs involved in signaling networks and their potential as therapeutic targets or noninvasive alternatives for screening to traditional methods for GI cancers (Table 2 and Table S1–S4).

## 6. The Potential Role of ncRNAs to GI Cancer Disparities

Race and ethnicity have long been associated with GI cancer health disparities. CRC incidence and mortality are highest in African Americans (AAs), followed closely by American Indians and Alaska Natives, and lowest in Asians/Pacific Islanders (APIs). During 2012–2016, CRC incidence rates in AAs were about 20% higher than those in Caucasian Americans (CAs) and 50% higher than those in APIs. The disparity for mortality is twice that for incidence; CRC death rates in AAs are almost 40% higher than those in CAs and double those in APIs [110]. Therefore, research is critically needed to understand these disparities and develop interventions to close the gap.

Single nucleotide polymorphisms (SNPs) located in the miRNA functional regions are involved in GI cancer susceptibility, often in a race-specific manner. Over a decade ago, it was revealed in two CRC prognostic research studies that the effect of SNP rs4919510 in miR-608 varied by race [111]. In CAs, the homozygous-variant genotype, GG, is associated with a significant increase in the risk of death, and in AAs, a protective association between the GG genotype and survival was observed [112]. Several studies have been carried out in Chinese populations investigating the association between genetic variants located in miRNAs and GI cancer susceptibility. SNPs rs2839698 in long noncoding RNA (lncRNA) gene H19 and rs2682818 in miR-618 were found to be associated with an elevated CRC risk, while miR-196a2 rs11614913 T > C polymorphism was shown to reduce the esophageal cancer risk in the Chinese study participants [113,114,115]. SNPs in miR-host genes (MIR17HG and MIR155HG) contributed to CRC and liver cancer susceptibility in Han Chinese populations [116,117,118]. Esophageal cancer patients were found to express an immune-related prognostic enhancer RNA, lncRNA AC007255.1, and gastric cancer patients expressed aberrant ncRNAs (miRNA-936, miRNA-1306-3p, miRNA-3185, miRNA- 6083, miRNA-659-3p, miRNA-6792-3p, lnc-ABCC5-2:1, lnc-MB21D1-3:5, and lnc-PSCA-4:2) in two recent studies from China [119,120]. One of the early studies to evaluate the prognostic value of miRNAs in CRC based on patient race/ethnicity demonstrated that miR-20a, miR-21, miR-106a, miR-181b, and miR-203 expressed two-fold higher in AA CRC patients than in their CA counterparts [121]. In 2016, a miR-1291-FOXA2-AGR2 signaling pathway was reported to control the suppression of pancreatic tumorigenesis in CA patients [122]. Oncogenic miRNAs (miR-17, miR-21, miR-182, miR-210, and miR-222) overexpressed in vitro in three newly established AA CRC lines, compared with the CA CRC lines [123]. When stratified by race (Asian and European), out of the 12 studies investigating polymorphisms in ncRNAs and susceptibility to CRC, only miR-146a rs2910164 was associated with a decreased risk of CRC in Europeans [124].

Several investigations have provided evidence supporting the role of the epigenetic regulation of miRNAs in racial cancer health disparities [125]. Hypermethylation of miR-9, miR-124, miR-137, miR-548, miR-663, miR-1207, miR-1279, miR-2682, miR-6130, and miR-182 was observed in AA CRC patients, while miR-34 was found to be hypermethylated in CA patients [126,127,128].

Some CRC racial disparities can be explained by differences in access to care, cancer screening, paucity of clinical data, and other socioeconomic factors [129,130]. However, reasons for ethnicity-based disparities are complex and remain even after adjustment for these factors. Consequently, a review of recent advances in the understanding of ethnicity-specific factors, including genetic, epigenetic, and environmental factors, related to tumorigenesis is important for evaluating our progress toward eliminating the disparities.

## 7. Discussion

GI cancers are common, both in the United States and worldwide. Early detection is still crucial for an effective treatment, which is however challenging due to the invasive screening methods or limited access to health care facilities. Recent advances in high-throughput sequencing technologies have revealed critical information about a variety of ncRNAs. Numerous reports have been documented to demonstrate the role of ncRNAs in tumor initiation and progression. The aberrant expression of ncRNAs has been observed to accompany DNA damage, immune escape, and cellular metabolic disorders in various cancer types, making it an interesting area of research to understand the pathogenesis of cancer. In this review, we highlighted the function of ncRNAs in modulating various cell signalizing pathways to induce GI cancer progression by epigenetic gene regulation, EMT, and development of drug resistance. However, it must be noted that, with the huge number of uncharacterized ncRNAs, the ncRNAs reviewed here are probably only a small proportion of the functionally relevant ncRNAs in GI cancer progression. Further, ncRNAs can be detected in plasma, have remarkably high tissue specificity, and are related to site-specific clinicopathological parameters including overall survival, recurrence, and metastasis; thus, they can be used as potential diagnostic and therapeutic markers in respective GI cancers. With the rapid development of gene-editing tools, the feasibility of CRISPR-Cas9-based ncRNA targeting in tumor cells is currently being explored [131,132]. However, the possibility of their off-target effects due to the low specificity of ncRNAs needs further validation. Other approaches such as combination therapy, with ncRNA-mediated targeted therapy using nanomedicine or immunotherapy, may be promising to treat GI cancers in the future. As early detection is still required to curb the spread of GI cancers and their efficient treatment, ncRNA-based screening tools could provide new noninvasive methods for GI cancer screening using patient blood/plasma samples. Finally, we noted the GI cancer health disparities and the predisposition of ncRNA in the respective ethnic groups. Conducting further studies to identify genetic markers in minority groups is crucial to reducing the mortality rate in these populations. In the United States, Hispanics are known to have a significantly higher incidence of GI cancers and worse cancer-related outcomes when compared with non-Hispanic white (NHW) patients. However, to the best of our knowledge, no ncRNA profiling studies have been conducted in this ethnic group. It is critical to study Hispanic GI cancers to identify potential ethnicity-specific biomarkers or targets for developing novel therapeutic interventions.

Taken together, ncRNA research has increased our understanding of the complexity of GI cancer progression and metastasis, although an understanding of their mechanistic function is only beginning to emerge. The major challenge remains the absence of appropriate therapeutic targets and detection systems for GI cancers, which could possibly be overcome by ncRNA-centered GI cancer research and their translation into clinical applications in the near future.

## Figures and Tables

**Table 1:** ncRNA-mediated chemoresistance targets in GI cancers.

ncRNA	GI Cancer Type	Expression	Drug(s)	Molecular Target(s)	Reference
lncRNA CRNDE	Colorectal cancer	Upregulated	5FU	*β-catenin* and *TCF4*	[24]
lncRNA PCAT6	Colorectal cancer	Upregulated	5FU	*HMGA2*	[49]
lncRNA SNHG6	Colorectal cancer	Upregulated	5FU	*ULK1*	[48]
miR-125b	Colorectal cancer	Downregulated	5FU	*APC*	[47]
miR-26a-5p	Colorectal cancer	Downregulated	5FU	*ULK1*	[48]
miR-328	Colorectal cancer	Upregulated	5FU and HCPT	*ABCG2*	[47]
miR-532-3p	Colorectal cancer	Upregulated	5FU, cisplatin	*ETS1* and *TGM2*	[47]
lncRNA HOTAIR	Colorectal cancer	Upregulated	cisplatin	*β-catenin, GRG5*	[23]
lncRNA CACS15	Colorectal cancer	Upregulated	oxaliplatin	*ABCC1*	[50]
lncRNA LINC00525	Colorectal cancer	Upregulated	oxaliplatin	*ELK3*	[51]
miR-128-3p	Colorectal cancer	Upregulated	oxaliplatin	*Bmi* and *MRP5*	[47]
lncRNA GIHCG	Colorectal cancer	Upregulated	oxaliplatin and 5FU	unknown	[52]
lncRNA SCARNA2	Colorectal cancer	Upregulated	oxaliplatin and 5FU	*WGFR* and *BCL-2*	[53]
miR-451	Colorectal cancer	Upregulated	SN38	*ABCB1*	[47]
miR-514b-3p	Colorectal cancer	Upregulated	cisplatin and irinotecan	*FZD4, NTN1*	[47]
miR-514b-5p	Colorectal cancer	Downregulated	cisplatin and irinotecan	*CDH1, CLDN1*	[47]
miR-138	Esophageal cancer	Downregulated	5FU and cisplatin	*Survivin*	[54]
lncRNA HOTAIR	Esophageal cancer	Upregulated	5FU	*MTHFR*	[55]
miR-221	Esophageal cancer	Downregulated	5FU	*DKK2*	[47]
miR-29c	Esophageal cancer	Downregulated	5FU	*FBXO3I*	[56]
miR-338-5p	Esophageal cancer	Downregulated	5FU	*ID-1*	[57]
lncRNA CCAT1	Esophageal cancer	Upregulated	cisplatin	*PLK1* and *BURB1*	[58]
lncRNA EMS	Esophageal cancer	Upregulated	cisplatin	*WTAP*	[59]
lncRNA LINC00337	Esophageal cancer	Upregulated	cisplatin	*TPX2*	[60]
lncRNA TUG1	Esophageal cancer	Upregulated	cisplatin	*NRF2*	[61]
miR-10b	Esophageal cancer	Downregulated	cisplatin	*PPARɣ*	[62]
lncRNA ROR	Gastric cancer	Upregulated	adriamycin and vincristine	*MRP1*	[63]
lncRNA HCP5	Gastric cancer	Upregulated	oxaliplatin and 5FU	*AMPK, PGC1α, CEBPB*	[64]
lncRNA ARHGAP5-AS1	Gastric cancer	Upregulated	cisplatin, actinomycin, and 5FU	*METTL3*	[65]
lncRNA SNHG16	Hepatocarcinoma	Downregulated	5FU	unknown	[66]
lncRNA CRNDE	Hepatocarcinoma	Upregulated	adriamycin	*CELF2* and *LATS2*	[46]
lncRNA GAS5	Hepatocarcinoma	Upregulated	doxorubicin	*PTEN*	[67]
lncRNA HANR	Hepatocarcinoma	Upregulated	doxorubicin	*GSKIP*	[68]
lncRNA MALAT1	Hepatocarcinoma	Upregulated	sorafenib	*miR-140-5p*	[69]
lncRNA H19	Hepatocarcinoma	Downregulated	sorafenib or doxorubicin	*ELAVL1*	[70]
miR-138-5p	Pancreatic cancer	Upregulated	5FU	*Vimentin*	[47]
miR-27b	Pancreatic cancer	Upregulated	docetaxel	*ZEB1*	[47]
miR-34a	Pancreatic cancer	Upregulated	docetaxel	*ZEB1*	[47]
lncRNA SLC7A11-AS1	Pancreatic cancer	Upregulated	gemcitabine	*NRF2*	[71]
miR-1243	Pancreatic cancer	Upregulated	gemcitabine	*SMAD4*	[47]
miR-153	Pancreatic cancer	Upregulated	gemcitabine	*Snail*	[47]
miR-30a	Pancreatic cancer	Upregulated	gemcitabine	*Snail*	[47]
miR-34	Pancreatic cancer	Upregulated	gemcitabine	*Slug*	[47]
miR-509-5p	Pancreatic cancer	Upregulated	gemcitabine	*Vimentin*	[47]
lncRNA GAS5	Pancreatic cancer	Upregulated	gemcitabine and 5FU	*MST-1*	[72]

**Table 2:** List of ncRNAs that could be used as therapeutic targets or biomarkers for colorectal cancer.

ncRNA	Expression	Clinical Significance	Molecular Target(s)	Reference
lncRNA TP73-AS1	Upregulated	Biomarker	*TGF-α*	[90]
lncRNA SH3PXD2A-AS1	Upregulated	Biomarker and therapeutic target	*P57* and *KLF2*	[91]
lncRNA RP1-85F18.6	Upregulated	Diagnostic or prognostic biomarker	cell proliferation, cell cycle progression, and apoptosis	[92]
lncRNA DLEU7-AS1	Upregulated	Prognostic biomarker	Wnt/β-Catenin signaling pathway	[93]
lncRNA BANCR	Upregulated	Prognostic biomarker	unknown	[94]
lncRNA HNF1A-AS1	Upregulated	Prognostic biomarker	Wnt/β-Catenin signaling pathway	[95]
lncRNA LUADT1	Upregulated	Prognostic biomarker	unknown	[96]
lncRNA PANDAR	Upregulated	Prognostic biomarker	unknown	[97]
lncRNA RP11-708H21.4	Downregulated	Prognostic biomarker	AKT/MTOR pathway	[98]
lncRNA SBDSP1	Upregulated	Prognostic biomarker	unknown	[99]
lncRNA SNHG6	Upregulated	Prognostic biomarker	cell proliferation, cell cycle progression, and apoptosis	[100]
lncRNA ZEB1-AS1	Upregulated	Prognostic biomarker	*P15*, *ZEB1*	[101]
lncRNA-RP11-317J10.2	Downregulated	Prognostic biomarker	*Cyclin D1*	[102]
lncRNAs (RP1-170O19.17, RP11-785D18.3, RP11-798K3.2, XXbac-B476C20.9, RP11-481J13.1, and RP11-167H9.4)	Upregulated	Prognostic biomarkers	cGMP-PKG signaling pathway and cAMP signaling pathway	[103]
lncRNA AB073614	Upregulated	Prognostic marker and therapeutic target	PI3K/AKT signaling pathway	[104]
lncRNA LINC00858	Upregulated	Prognostic marker and therapeutic target	miR-22-3p	[105]
lncRNA SNHG6	Upregulated	Prognostic marker and therapeutic target	*Upf1* and *ZEB1*	[106]
lncRNA CA3-AS1	Upregulated	Therapeutic target	miRNA-93/*PTEN* Axis	[107]
lncRNA Linc00659	Upregulated	Therapeutic target	PI3K/AKT signaling pathway	[108]
lncRNA-HOTAIR	Upregulated	Therapeutic target	*P21*	[109]

## Data Availability

Not applicable.

## Supplementary Materials

The following supporting information can be downloaded at: https://www.mdpi.com/article/10.3390/cells11152448/s1, Table S1: List of ncRNAs that could be used as therapeutic targets or biomarkers for gastric cancer [133,134,135,136,137,138,139,140,141,142,143,144,145,146,147,148,149,150,151,152,153,154,155,156,157,158,159,160,161,162,163,164,165,166,167,168,169]; Table S2: List of ncRNAs that could be used as therapeutic targets or biomarkers for pancreatic cancer [170,171,172,173,174,175,176,177,178,179,180,181,182,183,184,185,186,187,188,189,190,191,192,193,194,195,196,197,198,199,200]; Table S3: List of ncRNAs that could be used as therapeutic targets or biomarkers for liver cancer [201,202,203,204,205,206,207,208,209,210,211,212,213,214,215,216,217,218,219,220,221,222,223,224,225,226,227,228,229,230,231,232,233,234,235,236,237,238,239,240,241,242,243,244,245,246,247,248,249,250]; Table S4: List of ncRNAs that could be used as therapeutic targets or biomarkers for esophageal cancer [251,252,253,254,255,256,257,258,259,260,261,262,263,264,265,266,267,268,269,270,271,272,273,274,275,276,277,278,279,280,281,282,283,284,285,286,287,288,289].

## References

[1] Ferlay J., Colombet M., Soerjomataram I., Mathers C., Parkin D.M., Piñeros M., Znaor A., Bray F. (2019). Estimating the Global Cancer Incidence and Mortality in 2018: GLOBOCAN Sources and Methods. Int. J. Cancer.

[2] Sung H., Ferlay J., Siegel R.L., Laversanne M., Soerjomataram I., Jemal A., Bray F. (2021). Global Cancer Statistics 2020: GLOBOCAN Estimates of Incidence and Mortality Worldwide for 36 Cancers in 185 Countries. CA A Cancer J. Clin..

[3] Peery A.F., Crockett S.D., Murphy C.C., Jensen E.T., Kim H.P., Egberg M.D., Lund J.L., Moon A.M., Pate V., Barnes E.L. (2022). Burden and Cost of Gastrointestinal, Liver, and Pancreatic Diseases in the United States: Update 2021. Gastroenterology.

[4] Arnold M., Abnet C.C., Neale R.E., Vignat J., Giovannucci E.L., McGlynn K.A., Bray F. (2020). Global Burden of 5 Major Types of Gastrointestinal Cancer. Gastroenterology.

[5] Aznaourova M., Schmerer N., Schmeck B., Schulte L.N. (2020). Disease-Causing Mutations and Rearrangements in Long Non-Coding RNA Gene Loci. Front. Genet..

[6] Guttman M., Rinn J.L. (2012). Modular Regulatory Principles of Large Non-Coding RNAs. Nature.

[7] Sanchez Calle A., Kawamura Y., Yamamoto Y., Takeshita F., Ochiya T. (2018). Emerging Roles of Long Non-Coding RNA in Cancer. Cancer Sci..

[8] Romano G., Veneziano D., Acunzo M., Croce C.M. (2017). Small Non-Coding RNA and Cancer. Carcinogenesis.

[9] Esteller M. (2011). Non-Coding RNAs in Human Disease. Nat. Rev. Genet..

[10] Anastasiadou E., Jacob L.S., Slack F.J. (2017). Non-Coding RNA Networks in Cancer. Nat. Rev. Cancer.

[11] Zhang P., Wu W., Chen Q., Chen M. (2019). Non-Coding RNAs and Their Integrated Networks. J. Integr. Bioinform..

[12] Dai X., Kaushik A.C., Zhang J. (2019). The Emerging Role of Major Regulatory RNAs in Cancer Control. Front. Oncol..

[13] Derrien T., Johnson R., Bussotti G., Tanzer A., Djebali S., Tilgner H., Guernec G., Martin D., Merkel A., Knowles D.G. (2012). The GENCODE v7 Catalog of Human Long Noncoding RNAs: Analysis of Their Gene Structure, Evolution, and Expression. Genome Res.

[14] Brien J.O., Hayder H., Zayed Y., Peng C. (2018). Overview of MicroRNA Biogenesis, Mechanisms of Actions, and Circulation. Front. Endocrinol..

[15] Wang J., Liu X., Wu H., Ni P., Gu Z., Qiao Y., Chen N., Sun F., Fan Q. (2010). CREB Up-Regulates Long Non-Coding RNA, HULC Expression through Interaction with MicroRNA-372 in Liver Cancer. Nucleic Acids Res..

[16] Xiong J., Wang D., Wei A., Lu H., Tan C., Li A., Tang J., Wang Y., He S., Liu X. (2017). Deregulated Expression of MiR-107 Inhibits Metastasis of PDAC through Inhibition PI3K/Akt Signaling via Caveolin-1 and PTEN. Exp. Cell Res..

[17] Zhang Y.M., Wu Q.M., Chang L.Y., Liu J.C. (2020). MiR-34a and MiR-125a-5p Inhibit Proliferation and Metastasis but Induce Apoptosis in Hepatocellular Carcinoma Cells via Repressing the MACC1- Mediated PI3K/AKT/MTOR Pathway. Neoplasma.

[18] Zhao M.H., Liu W., Zhang Y., Liu J.J., Song H., Luo B. (2022). Epstein–Barr Virus MiR-BART4-3p Regulates Cell Proliferation, Apoptosis, and Migration by Targeting AXL in Gastric Carcinoma. Virus Genes.

[19] Brown J.M., Recht L., Strober S. (2017). The Promise of Targeting Macrophages in Cancer Therapy. Clin. Cancer Res..

[20] Wang D., Wang X., Si M., Yang J., Sun S., Wu H., Cui S., Qu X., Yu X. (2020). Exosome-Encapsulated MiRNAs Contribute to CXCL12/CXCR4-Induced Liver Metastasis of Colorectal Cancer by Enhancing M2 Polarization of Macrophages. Cancer Lett..

[21] Zhong M.E., Chen Y., Zhang G., Xu L., Ge W., Wu B. (2019). LncRNA H19 Regulates PI3K-Akt Signal Pathway by Functioning as a CeRNA and Predicts Poor Prognosis in Colorectal Cancer: Integrative Analysis of Dysregulated NcRNA-Associated CeRNA Network. Cancer Cell Int..

[22] Zhou S., Guo Z., Zhou C., Zhang Y., Wang S. (2021). Circ_NRIP1 Is Oncogenic in Malignant Development of Esophageal Squamous Cell Carcinoma (ESCC) via MiR-595/SEMA4D Axis and PI3K/AKT Pathway. Cancer Cell Int..

[23] Xiao Z., Qu Z., Chen Z., Fang Z., Zhou K., Huang Z., Guo X., Zhang Y. (2018). LncRNA HOTAIR Is a Prognostic Biomarker for the Proliferation and Chemoresistance of Colorectal Cancer via MiR-203a-3p- Mediated Wnt/ß-Catenin Signaling Pathway. Cell Physiol. Biochem..

[24] Han P., Li J., Zhang B., Lv J., Li Y., Gu X., Yu Z., Jia Y., Bai X. (2017). The LncRNA CRNDE Promotes Colorectal Cancer Cell Proliferation and Chemoresistance via MiR-181a-5p-Mediated Regulation of Wnt/β-Catenin Signaling. Mol. Cancer.

[25] Zhang J., Zhang K., Hou Y. (2020). Long Non-Coding RNA NNT-AS1 Knockdown Represses the Progression of Gastric Cancer via Modulating the MiR-142-5p/SOX4/Wnt/β-Catenin Signaling Pathway. Mol. Med. Rep..

[26] Peng Y., Qin Y., Zhang X., Deng S., Yuan Y., Feng X., Chen W., Hu F., Gao Y., He J. (2021). MiRNA-20b/SUFU/Wnt Axis Accelerates Gastric Cancer Cell Proliferation, Migration and EMT. Heliyon.

[27] Dong M., Gong L.-P., Chen J.-N., Zhang X.-F., Zhang Y.-W., Hui D.-Y., Zhao X.-X., Wu X.-Y., Shao C.-K. (2020). EBV-MiR-BART10-3p and EBV-MiR-BART22 Promote Metastasis of EBV-Associated Gastric Carcinoma by Activating the Canonical Wnt Signaling Pathway. Cell. Oncol..

[28] Cao X., Zhang J., Apaer S., Yao G., Li T. (2021). Microrna-19a-3p and Microrna-376c-3p Promote Hepatocellular Carcinoma Progression through Sox6-Mediated Wnt/β-Catenin Signaling Pathway. Int. J. Gen. Med..

[29] Peng Y., Zhang X., Lin H., Deng S., Huang Y., Qin Y., Feng X., Yan R., Zhao Y., Cheng Y. (2018). Inhibition of MiR-194 Suppresses the Wnt/β-Catenin Signalling Pathway in Gastric Cancer. Oncol. Rep..

[30] Hu Z., Wang P., Lin J., Zheng X., Yang F., Zhang G., Chen D., Xie J., Gao Z., Peng L. (2018). MicroRNA-197 Promotes Metastasis of Hepatocellular Carcinoma by Activating Wnt/β-Catenin Signaling. Cell. Physiol. Biochem..

[31] Song G.L., Xiao M., Wan X.Y., Deng J., Ling J.D., Tian Y.G., Li M., Yin J., Zheng R.Y., Tang Y. (2021). MiR-130a-3p Suppresses Colorectal Cancer Growth by Targeting Wnt Family Member 1 (WNT1). Bioengineered.

[32] Bai F., Zuo C., Ouyang Y., Xiao K., He Z., Yang Z. (2022). Circular RNA 0001666 Inhibits Colorectal Cancer Cell Proliferation, Invasion and Stemness by Inactivating the Wnt/Β-catenin Signaling Pathway and Targeting MicroRNA-1229. Oncol. Lett..

[33] Moustakas A., Pardali K., Gaal A., Heldin C.-H. (2002). Mechanisms of TGF-β Signaling in Regulation of Cell Growth and Differentiation. Immunol. Lett..

[34] Zhang J., Zhu Y., Hu L., Yan F., Chen J. (2019). MiR-494 Induces EndMT and Promotes the Development of HCC (Hepatocellular Carcinoma) by Targeting SIRT3/TGF-β/SMAD Signaling Pathway. Sci. Rep..

[35] Zhang L., Cai Q.Y., Liu J., Peng J., Chen Y.Q., Sferra T.J., Lin J.M. (2019). Ursolic Acid Suppresses the Invasive Potential of Colorectal Cancer Cells by Regulating the TGF-Β1/ZEB1/MiR-200c Signaling Pathway. Oncol. Lett..

[36] Yu D., Liu H., Qin J., Huangfu M., Guan X., Li X., Zhou L., Dou T., Liu Y., Wang L. (2021). Curcumol Inhibits the Viability and Invasion of Colorectal Cancer Cells via MiR-30a-5p and Hippo Signaling Pathway. Oncol. Lett..

[37] Xu X., Chen X., Xu M., Liu X., Pan B., Qin J., Xu T., Zeng K., Pan Y., He B. (2019). MiR-375-3p Suppresses Tumorigenesis and Partially Reverses Chemoresistance by Targeting YAP1 and SP1 in Colorectal Cancer Cells. Aging.

[38] Wang X., Chen Y., Liu W., Liu T., Sun D. (2020). Hsa_circ_0128846 Promotes Tumorigenesis of Colorectal Cancer by Sponging Hsa-MiR-1184 and Releasing AJUBA and Inactivating Hippo/YAP Signalling. J. Cell. Mol. Med..

[39] Huang F.-T., Peng J.-F., Cheng W.-J., Zhuang Y.-Y., Li C.-Q., Tang J., Chen W.-Y., Li Y.-H., Zhang S.-N. (2017). MiR-143 Targeting TAK1 Attenuates Pancreatic Ductal Adenocarcinoma Progression via MAPK and NF-JB Pathway In Vitro. Dig. Dis. Sci..

[40] Li Z., Lu J., Zeng G., Pang J., Zheng X., Feng J., Zhang J. (2019). MiR-129-5p Inhibits Liver Cancer Growth by Targeting Calcium Calmodulin-Dependent Protein Kinase IV (CAMK4). Cell Death Dis..

[41] Li Y., Wu D., Wang P., Li X., Shi G. (2017). MiR-195 Regulates Proliferation and Apoptosis through Inhibiting the MTOR/P70s6k Signaling Pathway by Targeting HMGA2 in Esophageal Carcinoma Cells. Dis. Markers.

[42] Zhao D.-W., Hou Y.-S., Sun F.-B., Han B., Li S.-J. (2019). Effects of MiR-132 on Proliferation and Apoptosis of Pancreatic Cancer Cells via Hedgehog Signaling Pathway. Eur. Rev. Med. Pharmacol. Sci..

[43] Chen Y., Zhang R. (2021). Long Non-Coding RNA AL139002.1 Promotes Gastric Cancer Development by Sponging MicroRNA-490-3p to Regulate Hepatitis A Virus Cellular Receptor 1 Expression. Bioengineered.

[44] Bukowski K., Kciuk M., Kontek R. (2020). Mechanisms of Multidrug Resistance in Cancer Chemotherapy1. Bukowski, K.; Kciuk, M.; Kontek, R. Mechanisms of Multidrug Resistance in Cancer Chemotherapy. Int. J. Mol. Sci..

[45] Zhang F., Wang H., Yu J., Yao X., Yang S., Li W., Xu L., Zhao L. (2021). LncRNA CRNDE Attenuates Chemoresistance in Gastric Cancer via SRSF6-Regulated Alternative Splicing of PICALM. Mol. Cancer.

[46] Xie S., Zhang J., Jiang X., Hua Y., Xie S., Qin Y., Yang Y. (2020). LncRNA CRNDE Facilitates Epigenetic Suppression of CELF2 and LATS2 to Promote Proliferation, Migration and Chemoresistance in Hepatocellular Carcinoma. Cell Death Dis..

[47] Dong B., Li S., Zhu S., Yi M., Luo S., Wu K. (2021). MiRNA-Mediated EMT and CSCs in Cancer Chemoresistance. Exp. Hematol. Oncol..

[48] Wang X., Lan Z., He J., Lai Q., Yao X., Li Q., Liu Y., Lai H., Gu C., Yan Q. (2019). LncRNA SNHG6 Promotes Chemoresistance through ULK1-Induced Autophagy by Sponging MiR-26a-5p in Colorectal Cancer Cells. Cancer Cell Int..

[49] Wu H., Fan D. (2019). Long Non-Coding RNA PCAT6 Targets MiR-204 to Modulate the Chemoresistance of Colorectal Cancer Cells to 5-Fluorouracil-Based Treatment through HMGA2 Signaling. Cancer Med..

[50] Gao R., Fang C., Xu J., Tan H., Li P., Ma L. (2019). LncRNA CACS15 Contributes to Oxaliplatin Resistance in Colorectal Cancer by Positively Regulating ABCC1 through Sponging MiR-145. Arch. Biochem. Biophys..

[51] Wang S., Li J., Yang X. (2019). Long Non-Coding RNA LINC00525 Promotes the Stemness and Chemoresistance of Colorectal Cancer by Targeting MiR-507 / ELK3 Axis. Int. J. Stem Cells.

[52] Jiang X., Li Q., Zhang S., Song C., Zheng P. (2019). Long Noncoding RNA GIHCG Induces Cancer Progression and Chemoresistance and Indicates Poor Prognosis in Colorectal Cancer. Onco Targets Ther..

[53] Zhang P.F., Liu M., Wang F. (2018). The LncRNA SCARNA2 Mediates Colorectal Cancer Chemoresistance through a Conserved MicroRNA-342-3p Target Sequence. J. Cell. Physiol..

[54] Wu J., Wang L., Du X., Sun Q., Wang Y., Li M., Zang W., Liu K., Zhao G. (2018). α-Solanine Enhances the Chemosensitivity of Esophageal Cancer Cells by Inducing MicroRNA-138 Expression. Oncol. Rep..

[55] Zhang S., Zheng F., Zhang L., Huang Z., Huang X., Pan Z. (2020). LncRNA HOTAIR-Mediated MTHFR Methylation Inhibits 5-Fluorouracil Sensitivity in Esophageal Cancer Cells. J. Exp. Clin. Cancer Res..

[56] Li B., Hong P., Zheng C.-C., Dai W., Chen W.-Y., Yang Q.-S., Han L., Tsao S.W., Chan K.T., Lee N.P.Y. (2019). Identification of MiR-29c and Its Target FBXO31 as a Key Regulatory Mechanism in Esophageal Cancer Chemoresistance: Functional Validation and Clinical Significance. Theranostics.

[57] Han L., Cui D., Li B., Xu W.W., Lam A.K.Y., Chan K.T., Zhu Y., Lee N.P.Y., Law S.Y.K., Guan X.Y. (2019). MicroRNA-338-5p Reverses Chemoresistance and Inhibits Invasion of Esophageal Squamous Cell Carcinoma Cells by Targeting Id-1. Cancer Sci..

[58] Hu M., Wang J.L. (2019). LncRNA CCAT1 Is a Biomarker for the Proliferation and Drug Resistance of Esophageal Cancer via the MiR -143/PLK1/BUBR1 Axis. Mol. Carcinog..

[59] Zhu Z., Pang Y., Jin G., Zhang H., Wang W., Liu J., Tuo G., Wu P., Yang Y., Wang Z. (2021). Hypoxia Induces Chemoresistance of Esophageal Cancer Cells to Cisplatin through Regulating the LncRNA-EMS/MiR-758-3p/WTAP Axis. Aging.

[60] Yang C., Lu Y. (2020). Long Non-Coding RNA LINC00337 Induces Autophagy and Chemoresistance to Cisplatin in Esophageal Squamous Cell Carcinoma Cells via Upregulation of TPX2 by Recruiting E2F4. FASEB J..

[61] Zhang Z., Xiong R., Li C., Xu M., Guo M. (2019). LncRNA TUG1 Promotes Cisplatin Resistance in Esophageal Squamous Cell Carcinoma Cells by Regulating Nrf2. Acta Biochim. Biophys. Sin..

[62] Wu K., Hu Y., Yan K., Qi Y., Zhang C., Zhu D., Liu D., Zhao S. (2020). MicroRNA-10b Confers Cisplatin Resistance by Activating AKT/MTOR/P70S6K Signaling via Targeting PPARγ in Esophageal Cancer. J. Cell. Physiol..

[63] Wang S., Chen W., Yu H., Song Z., Li Q., Shen X., Wu Y., Zhu L., Ma Q., Xing D. (2020). LncRNA ROR Promotes Gastric Cancer Drug Resistance. Cancer Control.

[64] Wu H., Liu B., Chen Z., Li G., Zhang Z. (2020). MSC-Induced LncRNA HCP5 Drove Fatty Acid Oxidation through MiR-3619-5p/AMPK/PGC1 α/CEBPB Axis to Promote Stemness and Chemo- Resistance of Gastric Cancer. Cell Death Dis..

[65] Zhu L., Zhu Y., Han S., Chen M., Song P., Dai D., Xu W., Jiang T., Feng L., Shin V.Y. (2019). Impaired Autophagic Degradation of LncRNA ARHGAP5-AS1 Promotes Chemoresistance in Gastric Cancer. Cell Death Dis..

[66] Xu F., Zha G., Wu Y., Cai W., Ao J. (2018). Overexpressing LncRNA SNHG16 Inhibited HCC Proliferation and Chemoresistance by Functionally Sponging Hsa-MiR-93. Onco Targets Ther..

[67] Wang C., Ke S., Li M., Lin C., Liu X., Pan Q. (2020). Downregulation of LncRNA GAS5 Promotes Liver Cancer Proliferation and Drug Resistance by Decreasing PTEN Expression. Mol. Genet. Genom..

[68] Xiao J., Lv Y., Jin F., Liu Y., Ma Y., Xiong Y., Liu L., Zhang S., Sun Y., Tipoe G.L. (2017). LncRNA HANR Promotes Tumorigenesis and Increase of Chemoresistance in Hepatocellular Carcinoma. Cell. Physiol. Biochem..

[69] Fan L., Huang X., Chen J., Zhang K., Gu Y.-H., Sun J., Cui S.-Y. (2020). Long Noncoding RNA MALAT1 Contributes to Sorafenib Resistance by Targeting MiR-140-5p/Aurora-A Signaling in Hepatocellular Carcinoma. Mol. Cancer Ther..

[70] Schultheiss C.S., Laggai S., Czepukojc B., Hussein U.K., List M., Tierling S., Hosseini K., Golob-schwarzl N., Pokorny J., Hachenthal N. (2017). The Long Non-Coding RNA H19 Suppresses Carcinogenesis and Chemoresistance in Hepatocellular Carcinoma. Cell Stress.

[71] Yang Q., Li K., Huang X., Zhao C., Mei Y., Li X., Jiao L., Yang H. (2020). LncRNA SLC7A11-AS1 Promotes Chemoresistance by Blocking SCF b -TRCP -Mediated Degradation of NRF2 in Pancreatic Cancer. Mol. Ther. Nucleic Acid.

[72] Gao Z., Wang J., Chen D., Ma X., Yang W., Zhe T., Dang X. (2018). Biomedicine & Pharmacotherapy Long Non-Coding RNA GAS5 Antagonizes the Chemoresistance of Pancreatic Cancer Cells through down-Regulation of MiR-181c-5p. Biomed. Pharmacother..

[73] Shimura T., Toden S., Kandimalla R., Toiyama Y., Okugawa Y., Kanda M., Baba H., Kodera Y., Kusunoki M., Goel A. (2021). Genomewide Expression Profiling Identifies a Novel MiRNA-Based Signature for the Detection of Peritoneal Metastasis in Patients with Gastric Cancer. Ann. Surg..

[74] Kalmár A., Nagy Z.B., Galamb O., Csabai I., Bodor A., Wichmann B., Valcz G., Barták B.K., Tulassay Z., Igaz P. (2019). Genome-Wide Expression Profiling in Colorectal Cancer Focusing on LncRNAs in the Adenoma-Carcinoma Transition. BMC Cancer.

[75] Chen D., Sun Q., Cheng X., Zhang L., Song W., Zhou D., Lin J., Wang W. (2016). Genome-Wide Analysis of Long Noncoding RNA (LncRNA) Expression in Colorectal Cancer Tissues from Patients with Liver Metastasis. Cancer Med..

[76] Yao J., Wu L., Meng X., Yang H., Ni S., Wang Q., Zhou J., Zhang Q., Su K., Shao L. (2017). Profiling, Clinicopathological Correlation and Functional Validation of Specific Long Non-Coding RNAs for Hepatocellular Carcinoma. Mol. Cancer.

[77] Xue J., Jia E., Ren N., Xin H. (2021). Identification of Prognostic MiRNA Biomarkers for Esophageal Cancer Based on The Cancer Genome Atlas and Gene Expression Omnibus. Medicine.

[78] Chen F., Zhou H., Wu C., Yan H. (2018). Identification of MiRNA Profiling in Prediction of Tumor Recurrence and Progress and Bioinformatics Analysis for Patients with Primary Esophageal Cancer: Study Based on TCGA Database. Pathol. Res. Pract..

[79] Ma J., Xiao Y., Tian B., Chen S., Zhang B., Wu J., Wu Z., Li X., Tang J., Yang D. (2019). Genome-Wide Analyses of Long Non-Coding RNA Expression Profiles and Functional Network Analysis in Esophageal Squamous Cell Carcinoma. Sci. Rep..

[80] Cui H., Zhang Y., Zhang Q., Chen W., Zhao H., Liang J. (2017). A Comprehensive Genome-Wide Analysis of Long Noncoding RNA Expression Profile in Hepatocellular Carcinoma. Cancer Med..

[81] Zheng R., Liang J., Lu J., Li S., Zhang G., Wang X., Liu M., Wang W., Chu H., Tao G. (2019). Genome-Wide Long Non-Coding RNAs Identified a Panel of Novel Plasma Biomarkers for Gastric Cancer Diagnosis. Gastric Cancer.

[82] Zhang K., Shi H., Xi H., Wu X., Cui J., Gao Y., Liang W., Hu C., Liu Y., Li J. (2017). Genome-Wide LncRNA Microarray Profiling Identifies Novel Circulating LncRNAs for Detection of Gastric Cancer. Theranostics.

[83] Lin P., Wen D.Y., Li Q., He Y., Yang H., Chen G. (2018). Genome-Wide Analysis of Prognostic LncRNAs, MiRNAs, and MRNAs Forming a Competing Endogenous RNA Network in Hepatocellular Carcinoma. Cell. Physiol. Biochem..

[84] Xie J., Chen L., Sun Q., Li H., Wei W., Wu D., Hu Y., Zhu Z., Shi J., Wang M. (2022). An Immune Subtype-Related Prognostic Signature of Hepatocellular Carcinoma Based on Single-Cell Sequencing Analysis. Aging.

[85] Pavlova N.N., Thompson C.B. (2016). The Emerging Hallmarks of Cancer Metabolism. Cell Metab..

[86] Mujica M.L., Gallay P.A., Perrachione F., Montemerlo A.E., Tamborelli L.A., Vaschetti V.M., Reartes D.F., Bollo S., Rodríguez M.C., Dalmasso P.R. (2020). New Trends in the Development of Electrochemical Biosensors for the Quantification of MicroRNAs. J. Pharm. Biomed. Anal..

[87] Zhou X., Yin C., Dang Y., Ye F., Zhang G. (2015). Identification of the Long Non-Coding RNA H19 in Plasma as a Novel Biomarker for Diagnosis of Gastric Cancer. Sci. Rep..

[88] Toden S., Zumwalt T.J., Goel A. (2021). Non-Coding RNAs and Potential Therapeutic Targeting in Cancer. Biochim. Biophys. Acta-Rev. Cancer.

[89] Chandra Gupta S., Nandan Tripathi Y. (2017). Potential of Long Non-Coding RNAs in Cancer Patients: From Biomarkers to Therapeutic Targets. Int. J. Cancer.

[90] Cai Y., Yan P., Zhang G., Yang W., Wang H., Cheng X. (2018). Long Non-Coding RNA TP73-AS1 Sponges MiR-194 to Promote Colorectal Cancer Cell Proliferation, Migration and Invasion via up-Regulating TGFα. Cancer Biomark..

[91] Ma Z., Peng P., Zhou J., Hui B., Ji H., Wang J., Wang K. (2018). Long Non-Coding RNA SH3PXD2A-AS1 Promotes Cell Progression Partly through Epigenetic Silencing P57 and KLF2 in Colorectal Cancer. Cell. Physiol. Biochem..

[92] Ma Y., Chen Y., Lin C., Hu G. (2018). Biological Functions and Clinical Significance of the Newly Identified Long Non-coding RNA RP1-85F18.6 in Colorectal Cancer. Oncol. Rep..

[93] Liu X.-B., Han C., Sun C.-Z. (2018). Long Non-Coding RNA DLEU7-AS1 Promotes the Occurrence and Development of Colorectal Cancer via Wnt/β-Catenin Pathway. Eur. Rev. Med. Pharmacol. Sci..

[94] Shen X., Bai Y., Luo B., Zhou X. (2017). Upregulation of LncRNA BANCR Associated with the Lymph Node Metastasis and Poor Prognosis in Colorectal Cancer. Biol. Res..

[95] Zhang X., Xiong Y., Tang F., Bian Y., Chen Y., Zhang F. (2017). Long Noncoding RNA HNF1A-AS1 Indicates a Poor Prognosis of Colorectal Cancer and Promotes Carcinogenesis via Activation of the Wnt/β-Catenin Signaling Pathway. Biomed. Pharmacother..

[96] Zhang X.F., Zhang Y., Shen Z., Yang G.G., Wang H.D., Li L.F., Liu D.C., Qiu J.M. (2018). LncRNALUADT1 Is Overexpressed in Colorectal Cancer and Its Expression Level Is Related to Clinicopathology. Eur. Rev. Med. Pharm. Sci..

[97] Li X., Wang F., Sun Y., Fan Q., Cui G. (2017). Expression of Long Non-Coding RNA PANDAR and Its Prognostic Value in Colorectal Cancer Patients. Int. J. Biol. Markers.

[98] Sun L., Jiang C., Xu C., Xue H., Zhou H., Gu L., Liu Y., Xu Q. (2017). Down-Regulation of Long Non-Coding RNA RP11-708H21.4 Is Associated with Poor Prognosis for Colorectal Cancer and Promotes Tumorigenesis through Regulating AKT/MTOR Pathway. Oncotarget.

[99] Zhou H.-B., Li Q., Liu M., Cao Y.-Q., Xu J.-Y. (2017). Increased Expression of Long Non-Coding RNA SBDSP1 Correlates with Poor Survival in Colorectal Cancer. Eur. Rev. Med. Pharmacol. Sci..

[100] Li M., Bian Z., Yao S., Zhang J., Jin G., Wang X., Yin Y., Huang Z. (2018). Up-Regulated Expression of SNHG6 Predicts Poor Prognosis in Colorectal Cancer. Pathol. Res. Pract..

[101] Fu J., Cui Y. (2017). Long Noncoding RNA ZEB1-AS1 Expression Predicts Progression and Poor Prognosis of Colorectal Cancer. Int. J. Biol. Markers.

[102] Luo J., Xu L.-N., Zhang S.-J., Jiang Y.-G., Zhuo D.-X., Wu L.-H., Jiang X., Huang Y. (2018). Downregulation of LncRNA-RP11-317J10.2 Promotes Cell Proliferation and Invasion and Predicts Poor Prognosis in Colorectal Cancer. Scand. J. Gastroenterol..

[103] Fan Q., Liu B. (2018). Discovery of a Novel Six-Long Non-Coding RNA Signature Predicting Survival of Colorectal Cancer Patients. J. Cell. Biochem..

[104] Wang Y., Kuang H., Xue J., Liao L., Yin F., Zhou X. (2017). LncRNA AB073614 Regulates Proliferation and Metastasis of Colorectal Cancer Cells via the PI3K/AKT Signaling Pathway. Biomed. Pharmacother..

[105] Sha Q.-K., Chen L., Xi J.-Z., Song H. (2019). Long Non-Coding RNA LINC00858 Promotes Cells Proliferation, Migration and Invasion by Acting as a CeRNA of MiR-22-3p in Colorectal Cancer. Artif. Cells Nanomed. Biotechnol..

[106] Wang X., Lai Q., He J., Li Q., Ding J., Lan Z., Gu C., Yan Q., Fang Y., Zhao X. (2019). LncRNA SNHG6 Promotes Proliferation, Invasion and Migration in Colorectal Cancer Cells by Activating TGF-β/Smad Signaling Pathway via Targeting UPF1 and Inducing EMT via Regulation of ZEB1. Int. J. Med. Sci..

[107] Wei H., Yang Z., Lin B. (2019). Overexpression of Long Non Coding RNA CA3-AS1 Suppresses Proliferation, Invasion and Promotes Apoptosis via MiRNA-93/PTEN Axis in Colorectal Cancer. Gene.

[108] Tsai K.-W., Lo Y.-H., Liu H., Yeh C.-Y., Chen Y.-Z., Hsu C.-W., Chen W.-S., Wang J.-H. (2018). Linc00659, a Long Noncoding RNA, Acts as Novel Oncogene in Regulating Cancer Cell Growth in Colorectal Cancer. Mol. Cancer.

[109] Lin K., Jiang H., Zhang L.-L., Jiang Y., Yang Y.-X., Qiu G.-D., She Y.-Q., Zheng J.-T., Chen C., Fang L. (2018). Down-Regulated LncRNA-HOTAIR Suppressed Colorectal Cancer Cell Proliferation, Invasion, and Migration by Mediating P21. Dig. Dis. Sci..

[110] American Cancer Society (2020). Colorectal Cancer Facts & Figures.

[111] Xing J., Wan S., Zhou F., Qu F., Li B., Myers R.E., Fu X., Palazzo J.P., He X., Chen Z. (2012). Genetic Polymorphisms in Pre-MicroRNA Genes as Prognostic Markers of Colorectal Cancer. Cancer Epidemiol. Biomark. Prev..

[112] Ryan B.M., McClary A.C., Valeri N., Robinson D., Paone A., Bowman E.D., Robles A.I., Croce C., Harris C.C. (2012). Rs4919510 in Hsa-Mir-608 Is Associated with Outcome but Not Risk of Colorectal Cancer. PLoS ONE.

[113] Wei J., Zheng L., Liu S., Yin J., Wang L., Wang X., Shi Y., Shao A., Tang W., Ding G. (2013). MiR-196a2 Rs11614913 T>C Polymorphism and Risk of Esophageal Cancer in a Chinese Population. Hum. Immunol..

[114] Chen Y., Du M., Chen W., Zhu L., Wu C., Zhang Z., Wang M., Chu H., Gu D., Chen J. (2018). Polymorphism Rs2682818 in MiR-618 Is Associated with Colorectal Cancer Susceptibility in a Han Chinese Population. Cancer Med..

[115] Li S., Hua Y., Jin J., Wang H., Du M., Zhu L., Chu H., Zhang Z., Wang M. (2016). Association of Genetic Variants in LncRNA H19 with Risk of Colorectal Cancer in a Chinese Population. Oncotarget.

[116] Chao X., Feng X., Shi H., Wang Y., Wang L., Shen H., Zha Q., Chen Y., Jiang C. (2020). MIR17HG Polymorphism (Rs7318578) Is Associated with Liver Cancer Risk in the Chinese Han Population. Biosci. Rep..

[117] Wu H., He G., Han H., Xiong W., Song T., Chen H., Chen X., Wu X., Huang G., Zhang Y. (2019). Analysis of MIR155HG Variants and Colorectal Cancer Susceptibility in Han Chinese Population. Mol. Genet. Genom. Med..

[118] Chen P., Bai Y., Li Y., Yuan Y., Cheng Y., Pang J., Zhu H., Chen C. (2019). Association between Polymorphisms of MIR17HG and Risk of Colorectal Cancer in the Chinese Han Population. Mol. Genet. Genom. Med..

[119] Yu Z., Rong Z.Y., Sheng J., Luo Z., Zhang J., Li T., Zhu Z., Fu Z., Qiu Z., Huang C. (2021). Aberrant Non-Coding RNA Expressed in Gastric Cancer and Its Diagnostic Value. Front. Oncol..

[120] Wang Q., Yu X., Yang N., Xu L., Zhou Y. (2021). LncRNA AC007255.1, an Immune-Related Prognostic Enhancer RNA in Esophageal Cancer. PeerJ.

[121] Bovell L.C., Shanmugam C., Putcha B.D.K., Katkoori V.R., Zhang B., Bae S., Singh K.P., Grizzle W.E., Manne U. (2013). The Prognostic Value of MicroRNAs Varies with Patient Race/Ethnicity and Stage of Colorectal Cancer. Clin. Cancer Res..

[122] Tu M.J., Pan Y.Z., Qiu J.X., Kim E.J., Yu A.M. (2016). MicroRNA-1291 Targets the FOXA2-AGR2 Pathway to Suppress Pancreatic Cancer Cell Proliferation and Tumorigenesis. Oncotarget.

[123] Paredes J., Ji P., Lacomb J.F., Shroyer K.R., Martello L.A., Williams J.L. (2018). Establishment of Three Novel Cell Lines Derived from African American Patients with Colorectal Carcinoma: A Unique Tool for Assessing Racial Health Disparity. Int. J. Oncol..

[124] Alidoust M., Hamzehzadeh L., Rivandi M., Pasdar A. (2018). Polymorphisms in Non-Coding RNAs and Risk of Colorectal Cancer: A Systematic Review and Meta-Analysis. Crit. Rev. Oncol./Hematol..

[125] Ahmad A., Azim S., Zubair H., Khan M.A., Singh S., Carter J.E., Rocconi R.P., Singh A.P. (2017). Epigenetic Basis of Cancer Health Disparities: Looking beyond Genetic Differences. Biochim. Biophys. Acta-Rev. Cancer.

[126] Li E., Ji P., Ouyang N., Zhang Y., Wang X.Y., Rubin D.C., Davidson N.O., Bergamaschi R., Shroyer K.R., Burke S. (2014). Differential Expression of MiRNAs in Colon Cancer between African and Caucasian Americans: Implications for Cancer Racial Health Disparities. Int. J. Oncol..

[127] Farhana L., Antaki F., Anees M.R., Nangia-Makker P., Judd S., Hadden T., Levi E., Murshed F., Yu Y., van Buren E. (2016). Role of Cancer Stem Cells in Racial Disparity in Colorectal Cancer. Cancer Med..

[128] Wang X., Ji P., Zhang Y., LaComb J.F., Tian X., Li E., Williams J.L. (2016). Aberrant DNA Methylation: Implications in Racial Health Disparity. PLoS ONE.

[129] Augustus G.J., Ellis N.A. (2018). Colorectal Cancer Disparity in African Americans: Risk Factors and Carcinogenic Mechanisms. Am. J. Pathol..

[130] Jackson C.S., Oman M., Patel A.M., Vega K.J. (2016). Health Disparities in Colorectal Cancer among Racial and Ethnic Minorities in the United States. J. Gastrointest. Oncol..

[131] Goyal A., Myacheva K., Groß M., Klingenberg M., Duran Arqué B., Diederichs S. (2017). Challenges of CRISPR/Cas9 Applications for Long Non-Coding RNA Genes. Nucleic Acids Res..

[132] Said H., Sameh M., Shawky M., Meteini E. (2020). Genomics LncRNA- RP11-156p1.3, Novel Diagnostic and Therapeutic Targeting via CRISPR / Cas9 Editing in Hepatocellular Carcinoma. Genomics.

[133] Wang J.-J., Yang Y.-C., Song Y.-X., Peng G., Sun J.-X., Chen X.-W., Ma B., Wang Z.-N. (2018). Long Non-Coding RNA AB007962 Is Downregulated in Gastric Cancer and Associated with Poor Prognosis. Oncol. Lett..

[134] Sun Y.-Y., Zhang H., Ma R.-R., Zhang G.-H., Tian Y.-R., Liu L., Liu L., Gao P. (2020). Long Non-Coding RNA AK025387 Promotes Cell Migration and Invasion of Gastric Cancer. Front. Oncol..

[135] Luo X., Wang G.-H., Bian Z.-L., Li X.-W., Zhu B.-Y., Jin C.-J., Ju S.-Q. (2018). Long Non-Coding RNA CCAL/MiR-149/FOXM1 Axis Promotes Metastasis in Gastric Cancer. Cell Death Dis..

[136] Xiao K., Dong Z., Wang D., Liu M., Ding J., Chen W., Shang Z., Yue C., Zhang Y. (2021). Clinical Value of LncRNA CCAT1 in Serum Extracellular Vesicles as a Potential Biomarker for Gastric Cancer. Oncol. Lett..

[137] Hao Y.-P., Qiu J.-H., Zhang D.-B., Yu C.-G. (2017). Long Non-Coding RNA DANCR, a Prognostic Indicator, Promotes Cell Growth and Tumorigenicity in Gastric Cancer. Tumour Biol..

[138] Song W., Qian Y., Zhang M.-H., Wang H., Wen X., Yang X.-Z., Dai W.-J. (2020). The Long Non-Coding RNA DDX11-AS1 Facilitates Cell Progression and Oxaliplatin Resistance via Regulating MiR-326/IRS1 Axis in Gastric Cancer. Eur. Rev. Med. Pharmacol. Sci..

[139] Liang Y., Zhang C.-D., Zhang C., Dai D.-Q. (2020). DLX6-AS1/MiR-204-5p/OCT1 Positive Feedback Loop Promotes Tumor Progression and Epithelial-Mesenchymal Transition in Gastric Cancer. Gastric Cancer.

[140] Luo Y., Zheng S., Wu Q., Wu J., Zhou R., Wu Z., Rong X., Huang N., Sun L., Bin J. (2021). Long Noncoding RNA (LncRNA) EIF3J-DT Induces Chemoresistance of Gastric Cancer via Autophagy Activation. Autophagy.

[141] Zhang G., Wang Q., Lu J., Ma G., Ge Y., Chu H., Du M., Wang M., Zhang Z. (2019). Long Non-Coding RNA FLJ22763 Is Involved in the Progression and Prognosis of Gastric Cancer. Gene.

[142] Bo G., Liu Y., Li W., Wang L., Zhao L., Tong D., Ni L., Liu L., Qin Y., Wang W. (2022). The Novel LncRNA GPC5-AS1 Stabilizes GPC5 MRNA by Competitively Binding with MiR-93/106a to Suppress Gastric Cancer Cell Proliferation. Aging.

[143] Yörüker E.E., Keskin M., Kulle C.B., Holdenrieder S., Gezer U. (2018). Diagnostic and Prognostic Value of Circulating LncRNA H19 in Gastric Cancer. Biomed. Rep..

[144] Da M., Ma J., Zhang Y., Yang J., Yao J., Huang B., Ma H., Ge L. (2017). High Expression Level of Long Non-Coding RNA HOTAIR Is Associated with Poor Overall Survival in Gastric Cancer Patients: Evidence from Meta-Analysis. J. BUON.

[145] Yao L., Ye P.-C., Tan W., Luo Y.-J., Xiang W.-P., Liu Z.-L., Fu Z.-M., Lu F., Tang L.-H., Xiao J.-W. (2020). Decreased Expression of the Long Non-Coding RNA HOXD-AS2 Promotes Gastric Cancer Progression by Targeting HOXD8 and Activating PI3K/Akt Signaling Pathway. World J. Gastrointest. Oncol..

[146] Hu Z., Yang D., Tang Y., Zhang X., Wei Z., Fu H., Xu J., Zhu Z., Cai Q. (2019). Five-Long Non-Coding RNA Risk Score System for the Effective Prediction of Gastric Cancer Patient Survival. Oncol. Lett..

[147] Zhong X., Wen X., Chen L., Gu N., Yu X., Sui K. (2021). Long Non-Coding RNA KCNQ1OT1 Promotes the Progression of Gastric Cancer via the MiR-145-5p/ARF6 Axis. J. Gene Med..

[148] Wang H., Chen W., Yang P., Jun Z., Wang K., Tao Q. (2019). Knockdown of Linc00152 Inhibits the Progression of Gastric Cancer by Regulating MicroRNA-193b-3p/ETS1 Axis. Cancer Biol. Ther..

[149] He W., Zhang D., Li D., Zhu D., Geng Y., Wang Q., He J., Wu J. (2021). Knockdown of Long Non-Coding RNA LINC00200 Inhibits Gastric Cancer Progression by Regulating MiR-143-3p/SERPINE1 Axis. Dig. Dis. Sci..

[150] Li D., Yang M., Liao A., Zeng B., Liu D., Yao Y., Hu G., Xuanmin Z.C., Feng Z., Du Y. (2018). Linc00483 as CeRNA Regulates Proliferation and Apoptosis through Activating MAPKs in Gastric Cancer. J. Cell. Mol. Med..

[151] Pang K., Ran M.-J., Zou F.-W., Yang T.-W., He F. (2018). Long Non-Coding RNA LINC00857 Promotes Gastric Cancer Cell Proliferation and Predicts Poor Patient Survival. Oncol. Lett..

[152] Fang Y., Huang S., Han L., Wang S., Xiong B. (2021). Comprehensive Analysis of Peritoneal Metastasis Sequencing Data to Identify LINC00924 as a Prognostic Biomarker in Gastric Cancer. Cancer Manag. Res..

[153] Liu H., Wu N., Zhang Z., Zhong X., Zhang H., Guo H., Nie Y., Liu Y. (2019). Long Non-Coding RNA LINC00941 as a Potential Biomarker Promotes the Proliferation and Metastasis of Gastric Cancer. Front. Genet..

[154] Yang X.-Z., Cheng T.-T., He Q.-J., Lei Z.-Y., Chi J., Tang Z., Liao Q.-X., Zhang H., Zeng L.-S., Cui S.-Z. (2018). LINC01133 as CeRNA Inhibits Gastric Cancer Progression by Sponging MiR-106a-3p to Regulate APC Expression and the Wnt/β-Catenin Pathway. Mol. Cancer.

[155] Meng S., Dolo P.R., Guo P., Hong J., Li C., Zhu X., Zhou D. (2022). The Expression of Long Non-Coding RNA LINC01279 in Gastric Adenocarcinoma and Its Clinical Significance. Asian J. Surg..

[156] Li Y., Wu Z., Yuan J., Sun L., Lin L., Huang N., Bin J., Liao Y., Liao W. (2017). Long Non-Coding RNA MALAT1 Promotes Gastric Cancer Tumorigenicity and Metastasis by Regulating Vasculogenic Mimicry and Angiogenesis. Cancer Lett..

[157] Luo T., Zhao J., Lu Z., Bi J., Pang T., Cui H., Yang B., Li W., Wang Y., Wu S. (2018). Characterization of Long Non-Coding RNAs and MEF2C-AS1 Identified as a Novel Biomarker in Diffuse Gastric Cancer. Transl. Oncol..

[158] Zhong F., Zhu M., Gao K., Xu P., Yang H., Hu D., Cui D., Wang M., Xie X., Wei Y. (2018). Low Expression of the Long Non-Coding RNA NR_026827 in Gastric Cancer. Am. J. Transl. Res..

[159] Li J.-F., Li W.-H., Xue L.-L., Zhang Y. (2019). Long Non-Coding RNA PICART1 Inhibits Cell Proliferation by Regulating the PI3K/AKT and MAPK/ERK Signaling Pathways in Gastric Cancer. Eur. Rev. Med. Pharmacol. Sci..

[160] Chen H., Xu Z., Liu X., Gao Y., Wang J., Qian P., Yang B. (2018). Increased Expression of LncRNA RP11-397A15.4 in Gastric Cancer and Its Clinical Significance. Ann. Clin. Lab. Sci..

[161] Wu Q., Ma J., Wei J., Meng W., Wang Y., Shi M. (2021). LncRNA SNHG11 Promotes Gastric Cancer Progression by Activating the Wnt/β-Catenin Pathway and Oncogenic Autophagy. Mol. Ther..

[162] Liu Y.-Y., Chen Z.-H., Peng J.-J., Wu J.-L., Yuan Y.-J., Zhai E.-T., Cai S.-R., He Y.-L., Song W. (2017). Up-Regulation of Long Non-Coding RNA XLOC_010235 Regulates Epithelial-to-Mesenchymal Transition to Promote Metastasis by Associating with Snail1 in Gastric Cancer. Sci. Rep..

[163] Chai H., Sun C., Liu J., Sheng H., Zhao R., Feng Z. (2019). The Relationship between ZEB1-AS1 Expression and the Prognosis of Patients with Advanced Gastric Cancer Receiving Chemotherapy. Technol. Cancer Res. Treat..

[164] Wang F., Li X., Zhao X., Xue Y. (2020). Detection of a 5-CircRNA Signature to Improve Prognostic Prediction in Gastric Cancer. J. Investig. Med..

[165] Xian H.-P., Zhuo Z.-L., Sun Y.-J., Liang B., Zhao X.-T. (2018). Circulating Long Non-Coding RNAs HULC and ZNFX1-AS1 Are Potential Biomarkers in Patients with Gastric Cancer. Oncol. Lett..

[166] Jiao Y., Zhang L., Li J., He Y., Zhang X., Li J. (2021). Exosomal MiR-122-5p Inhibits Tumorigenicity of Gastric Cancer by Downregulating GIT1. Int. J. Biol. Mark..

[167] Sui M., Jiao A., Zhai H., Wang Y., Wang Y., Sun D., Li P. (2017). Upregulation of MiR-125b Is Associated with Poor Prognosis and Trastuzumab Resistance in HER2-Positive Gastric Cancer. Exp. Ther. Med..

[168] Azarbarzin S., Feizi M.A.H., Safaralizadeh R., Kazemzadeh M., Fateh A. (2017). The Value of MiR-383, an Intronic MiRNA, as a Diagnostic and Prognostic Biomarker in Intestinal-Type Gastric Cancer. Biochem. Genet..

[169] Schaalan M., Mohamed W., Fathy S. (2020). MiRNA-200c, MiRNA-139 and Ln RNA H19; New Predictors of Treatment Response in H-Pylori- Induced Gastric Ulcer or Progression to Gastric Cancer. Microb. Pathog..

[170] Liu L., Liu F.-B., Huang M., Xie K., Xie Q.-S., Liu C.-H., Shen M.-J., Huang Q. (2019). Circular RNA CiRS-7 Promotes the Proliferation and Metastasis of Pancreatic Cancer by Regulating MiR-7-Mediated EGFR/STAT3 Signaling Pathway. Hepatobiliary Pancreat. Dis. Int..

[171] Wong C.H., Lou U.K., Fung F.K.-C., Tong J.H.M., Zhang C.-H., To K.-F., Chan S.L., Chen Y. (2022). CircRTN4 Promotes Pancreatic Cancer Progression through a Novel CircRNA-MiRNA-LncRNA Pathway and Stabilizing Epithelial-Mesenchymal Transition Protein. Mol. Cancer.

[172] Ma L., Tian X., Guo H., Zhang Z., Du C., Wang F., Xie X., Gao H., Zhuang Y., Kornmann M. (2018). Long Noncoding RNA H19 Derived MiR-675 Regulates Cell Proliferation by down-Regulating E2F-1 in Human Pancreatic Ductal Adenocarcinoma. J. Cancer.

[173] Fu Z., Li G., Li Z., Wang Y., Zhao Y., Zheng S., Ye H., Luo Y., Zhao X., Wei L. (2017). Endogenous MiRNA Sponge LincRNA-ROR Promotes Proliferation, Invasion and Stem Cell-like Phenotype of Pancreatic Cancer Cells. Cell Death Discov..

[174] Wang L., Wang F., Na L., Yu J., Huang L., Meng Z.-Q., Chen Z., Chen H., Ming L.-L., Hua Y.-Q. (2018). LncRNA AB209630 Inhibits Gemcitabine Resistance Cell Proliferation by Regulating PI3K/AKT Signaling in Pancreatic Ductal Adenocarcinoma. Cancer Biomark..

[175] Yun Z., Meng F., Li S., Zhang P. (2021). Long Non-Coding RNA CERS6-AS1 Facilitates the Oncogenicity of Pancreatic Ductal Adenocarcinoma by Regulating the MicroRNA-15a-5p/FGFR1 Axis. Aging.

[176] Yong S., Yabin Y., Bing Z., Chuanrong Z., Dianhua G., Jianhuai Z., Weidong Y., Shuming W., Ling L. (2017). Reciprocal Regulation of DGCR5 and MiR-320a Affects the Cellular Malignant Phenotype and 5-FU Response in Pancreatic Ductal Adenocarcinoma. Oncotarget.

[177] Gao S., Cai Y., Zhang H., Hu F., Hou L., Xu Q. (2019). Long Noncoding RNA DLEU1 Aggravates Pancreatic Ductal Adenocarcinoma Carcinogenesis via the MiR-381/CXCR4 Axis. J. Cell. Physiol..

[178] Ye H., Zhou Q., Zheng S., Li G., Lin Q., Ye L., Wang Y., Wei L., Zhao X., Li W. (2018). FEZF1-AS1/MiR-107/ZNF312B Axis Facilitates Progression and Warburg Effect in Pancreatic Ductal Adenocarcinoma. Cell Death Dis..

[179] Sun Y., Zhu Q., Yang W., Shan Y., Yu Z., Zhang Q., Wu H. (2019). LncRNA H19/MiR-194/PFTK1 Axis Modulates the Cell Proliferation and Migration of Pancreatic Cancer. J. Cell. Biochem..

[180] Fu Z., Chen C., Zhou Q., Wang Y., Zhao Y., Zhao X., Li W., Zheng S., Ye H., Wang L. (2017). LncRNA HOTTIP Modulates Cancer Stem Cell Properties in Human Pancreatic Cancer by Regulating HOXA9. Cancer Lett..

[181] Yang M., Qin Q., Zhu J., Guo Y., Yin T., Wu H., Wang C. (2020). Long Noncoding RNA ITGB2-AS1 Promotes Growth and Metastasis through MiR-4319/RAF1 Axis in Pancreatic Ductal Adenocarcinoma. J. Cell. Physiol..

[182] Wu J., Sun S., Liao W., Chen E., Wang X., Song Y., Duan F., Deng W., Li S. (2021). LINC00460 Promotes Pancreatic Cancer Progression by Sponging MiR-491-5p. J. Gene Med..

[183] Ni C., Zheng K., Liu W., Ou Y., Li G., Jin G. (2022). LINC00483 Promotes Proliferation and Metastasis through the MiR-19a-3p/TBK1/MAPK Axis in Pancreatic Ductal Adenocarcinoma (PDAC). Ann. Transl. Med..

[184] Bi S., Wang Y., Feng H., Li Q. (2020). Long Noncoding RNA LINC00657 Enhances the Malignancy of Pancreatic Ductal Adenocarcinoma by Acting as a Competing Endogenous RNA on MicroRNA-433 to Increase PAK4 Expression. Cell Cycle.

[185] Lu W., Zhang H., Niu Y., Wu Y., Sun W., Li H., Kong J., Ding K., Shen H.-M., Wu H. (2017). Long Non-Coding RNA Linc00673 Regulated Non-Small Cell Lung Cancer Proliferation, Migration, Invasion and Epithelial Mesenchymal Transition by Sponging MiR-150-5p. Mol. Cancer.

[186] Xu M., Cui R., Ye L., Wang Y., Wang X., Zhang Q., Wang K., Dong C., Le W., Chen B. (2021). LINC00941 Promotes Glycolysis in Pancreatic Cancer by Modulating the Hippo Pathway. Mol. Ther. Nucleic Acids.

[187] Chen M., Zhang C., Liu W., Du X., Liu X., Xing B. (2022). Long Noncoding RNA LINC01234 Promotes Hepatocellular Carcinoma Progression through Orchestrating Aspartate Metabolic Reprogramming. Mol. Ther..

[188] Nai Y., Pan C., Hu X., Ma Y. (2020). LncRNA LUCAT1 Contributes to Cell Proliferation and Migration in Human Pancreatic Ductal Adenocarcinoma via Sponging MiR-539. Cancer Med..

[189] Liu P., Yang H., Zhang J., Peng X., Lu Z., Tong W., Chen J. (2017). The LncRNA MALAT1 Acts as a Competing Endogenous RNA to Regulate KRAS Expression by Sponging MiR-217 in Pancreatic Ductal Adenocarcinoma. Sci. Rep..

[190] Zhuo M., Yuan C., Han T., Cui J., Jiao F., Wang L. (2018). A Novel Feedback Loop between High MALAT-1 and Low MiR-200c-3p Promotes Cell Migration and Invasion in Pancreatic Ductal Adenocarcinoma and Is Predictive of Poor Prognosis. BMC Cancer.

[191] Sun Y., Wang P., Yang W., Shan Y., Zhang Q., Wu H. (2019). The Role of LncRNA MSC-AS1/MiR-29b-3p Axis-Mediated CDK14 Modulation in Pancreatic Cancer Proliferation and Gemcitabine-Induced Apoptosis. Cancer Biol. Ther..

[192] Luo Z., Yi Z.-J., Ou Z.-L., Han T., Wan T., Tang Y.-C., Wang Z.-C., Huang F.-Z. (2019). RELA/NEAT1/MiR-302a-3p/RELA Feedback Loop Modulates Pancreatic Ductal Adenocarcinoma Cell Proliferation and Migration. J. Cell. Physiol..

[193] Wang X., Li H., Lu X., Wen C., Huo Z., Shi M., Tang X., Chen H., Peng C., Fang Y. (2018). Melittin-Induced Long Non-Coding RNA NONHSAT105177 Inhibits Proliferation and Migration of Pancreatic Ductal Adenocarcinoma. Cell Death Dis..

[194] Wu L., Liu Y., Guo C., Shao Y. (2020). LncRNA OIP5-AS1 Promotes the Malignancy of Pancreatic Ductal Adenocarcinoma via Regulating MiR-429/FOXD1/ERK Pathway. Cancer Cell Int..

[195] Zhang Y., Ma H., Chen C. (2021). Long Non-coding RNA PCED1B-AS1 Promotes Pancreatic Ductal Adenocarcinoma Progression by Regulating the MiR-411-3p/HIF-1α Axis. Oncol. Rep..

[196] Liu W., Tang J., Zhang H., Kong F., Zhu H., Li P., Li Z., Kong X., Wang K. (2020). A Novel LncRNA PTTG3P/MiR-132/212-3p/FoxM1 Feedback Loop Facilitates Tumorigenesis and Metastasis of Pancreatic Cancer. Cell Death Discov..

[197] Sun J., Zhang P., Yin T., Zhang F., Wang W. (2020). Upregulation of LncRNA PVT1 Facilitates Pancreatic Ductal Adenocarcinoma Cell Progression and Glycolysis by Regulating MiR-519d-3p and HIF-1A. J. Cancer.

[198] Huang S., Li Y., Hu J., Li L., Liu Z., Guo H., Jiang B., Chen J., Junhe X.L., Xiang X. (2021). LncRNA PWAR6 Regulates Proliferation and Migration by Epigenetically Silencing YAP1 in Tumorigenesis of Pancreatic Ductal Adenocarcinoma. J. Cell. Mol. Med..

[199] Li N., Yang G., Luo L., Ling L., Wang X., Shi L., Lan J., Jia X., Zhang Q., Long Z. (2020). LncRNA THAP9-AS1 Promotes Pancreatic Ductal Adenocarcinoma Growth and Leads to a Poor Clinical Outcome via Sponging MiR-484 and Interacting with YAP. Clin. Cancer Res..

[200] Miao H., Lu J., Guo Y., Qiu H.., Zhang Y., Yao X., Li X., Lu Y. (2021). LncRNA TP73-AS1 Enhances the Malignant Properties of Pancreatic Ductal Adenocarcinoma by Increasing MMP14 Expression through MiRNA -200a Sponging. J. Cell. Mol. Med..

[201] Bai J., Yao B., Wang L., Sun L., Chen T., Liu R., Yin G., Qiuran X., Yang W. (2019). LncRNA A1BG-AS1 Suppresses Proliferation and Invasion of Hepatocellular Carcinoma Cells by Targeting MiR-216a-5p. J. Cell. Biochem..

[202] Zeng T., Wang D., Chen J., Tian Y., Cai X., Peng H., Zhu L., Huang A., Tang H. (2017). LncRNA-AF113014 Promotes the Expression of Egr2 by Interaction with MiR-20a to Inhibit Proliferation of Hepatocellular Carcinoma Cells. PLoS ONE.

[203] Wang F., Zhu L., Xue Q., Tang C., Tang W., Zhang N., Dai C., Chen Z. (2022). Novel LncRNA AL033381.2 Promotes Hepatocellular Carcinoma Progression by Upregulating PRKRA Expression. Oxid. Med. Cell. Longev..

[204] Zhao X., Liu Y., Yu S. (2017). Long Noncoding RNA AWPPH Promotes Hepatocellular Carcinoma Progression through YBX1 and Serves as a Prognostic Biomarker. Biochim. Biophys. Acta.

[205] Chen F., Bai G., Li Y., Feng Y., Wang L. (2017). A Positive Feedback Loop of Long Noncoding RNA CCAT2 and FOXM1 Promotes Hepatocellular Carcinoma Growth. Am. J. Cancer Res..

[206] Zhuang H., Cao G., Kou C., Li D. (2019). Overexpressed LncRNA CDKN2B-AS1 Is an Independent Prognostic Factor for Liver Cancer and Promotes Its Proliferation. J. BUON.

[207] Chao Y., Zhou D. (2019). LncRNA-D16366 Is a Potential Biomarker for Diagnosis and Prognosis of Hepatocellular Carcinoma. Med. Sci. Monit..

[208] Li B., Mao R., Liu C., Zhang W., Tang Y., Guo Z. (2018). LncRNA FAL1 Promotes Cell Proliferation and Migration by Acting as a CeRNA of MiR-1236 in Hepatocellular Carcinoma Cells. Life Sci..

[209] Chen F., Li Y., Li M., Wang L. (2019). Long Noncoding RNA GAS5 Inhibits Metastasis by Targeting MiR-182/ANGPTL1 in Hepatocellular Carcinoma. Am. J. Cancer Res..

[210] Yu J., Hong J.-F., Kang J., Liao L.-H., Li C.-D. (2017). Promotion of LncRNA HOXA11-AS on the Proliferation of Hepatocellular Carcinoma by Regulating the Expression of LATS1. Eur. Rev. Med. Pharmacol. Sci..

[211] Zhou J.-F., Shi Y.-T., Wang H.-G., Yang X.-Z., Wu S.-N. (2019). Overexpression of Long Noncoding RNA HOXC13-AS and Its Prognostic Significance in Hepatocellular Carcinoma. Eur. Rev. Med. Pharmacol. Sci..

[212] Xiong H., Ni Z., He J., Jiang S., Li X., He J., Gong W., Zheng L., Chen S., Li B. (2017). LncRNA HULC Triggers Autophagy via Stabilizing Sirt1 and Attenuates the Chemosensitivity of HCC Cells. Oncogene.

[213] Xiong H., Li B., He J., Zeng Y., Zhang Y., He F. (2017). LncRNA HULC Promotes the Growth of Hepatocellular Carcinoma Cells via Stabilizing COX-2 Protein. Biochem. Biophys. Res. Commun..

[214] Xu L.-C., Chen Q.-N., Liu X.-Q., Wang X.-M., Chang Q.-M., Pan Q., Wang L., Wang Y.-L. (2017). Up-Regulation of LINC00161 Correlates with Tumor Migration and Invasion and Poor Prognosis of Patients with Hepatocellular Carcinoma. Oncotarget.

[215] Gao J., Dai C., Yu X., Yin X.-B., Zhou F. (2020). Long Noncoding RNA LINC00324 Exerts Protumorigenic Effects on Liver Cancer Stem Cells by Upregulating Fas Ligand via PU Box Binding Protein. FASEB J..

[216] Yin Y.-Z., Zheng W.-H., Zhang X., Chen Y.-H., Tuo Y.-H. (2020). LINC00346 Promotes Hepatocellular Carcinoma Progression via Activating the JAK-STAT3 Signaling Pathway. J. Cell. Biochem..

[217] Wu J.-H., Tian X.-Y., An Q.-M., Guan X.-Y., Hao C.-Y. (2018). LINC00963 Promotes Hepatocellular Carcinoma Progression by Activating PI3K/AKT Pathway. Eur. Rev. Med. Pharmacol. Sci..

[218] Zheng Y.-L., Li L., Jia Y.-X., Zhang B.-Z., Li J.-C., Zhu Y.-H., Li M.-Q., He J.-Z., Zeng T.-T., Ban X.-J. (2019). LINC01554-Mediated Glucose Metabolism Reprogramming Suppresses Tumorigenicity in Hepatocellular Carcinoma via Downregulating PKM2 Expression and Inhibiting Akt/MTOR Signaling Pathway. Theranostics.

[219] Chen M.H., Qi B., Cai Q.Q., Sun J.W., Fu L.S., Kang C.L., Fan F., Ma M.Z., Wu X.Z. (2022). LncRNA LncAY Is Upregulated by Sulfatide via Myb/MEF2C Acetylation to Promote the Tumorigenicity of Hepatocellular Carcinoma Cells. Biochim. Biophys. Acta Gene Regul. Mech..

[220] Dong H., Zhang Y., Xu Y., Ma R., Liu L., Luo C., Jiang W. (2019). Downregulation of Long Non-Coding RNA MEG3 Promotes Proliferation, Migration, and Invasion of Human Hepatocellular Carcinoma Cells by Upregulating TGF-Β1. Acta Biochim. Biophys. Sin..

[221] Shen X., Ding Y., Lu F., Yuan H., Luan W. (2020). Long Noncoding RNA MIR4435-2HG Promotes Hepatocellular Carcinoma Proliferation and Metastasis through the MiR-22-3p/YWHAZ Axis. Am. J. Transl. Res..

[222] Kong Q., Liang C., Jin Y., Pan Y., Tong D., Kong Q., Zhou J. (2019). The LncRNA MIR4435-2HG Is Upregulated in Hepatocellular Carcinoma and Promotes Cancer Cell Proliferation by Upregulating MiRNA-487a. Cell. Mol. Biol. Lett..

[223] Wang H., Liang L., Dong Q., Huan L., He J., Li B., Yang C., Jin H., Lin C.W., Yu C. (2018). Long Noncoding RNA MiR503HG, a Prognostic Indicator, Inhibits Tumor Metastasis by Regulating the HNRNPA2B1/NF-ΚB Pathway in Hepatocellular Carcinoma. Theranostics.

[224] Liu J., Zhao S.-Y., Jiang Q., Qu Y., Huang X., Du J., Sun W., Ye Q. (2020). Long Noncoding RNA MYLK-AS1 Promotes Growth and Invasion of Hepatocellular Carcinoma through the EGFR/HER2-ERK1/2 Signaling Pathway. Int. J. Biol. Sci..

[225] Ling Z.-A., Xiong D.-D., Meng R.-M., Cen J.-M., Zhao N., Chen G., Li R.-L., Dang Y.-W. (2018). LncRNA NEAT1 Promotes Deterioration of Hepatocellular Carcinoma Based on in Vitro Experiments, Data Mining, and RT-QPCR Analysis. Cell. Physiol. Biochem..

[226] Fang L., Sun J., Pan Z., Song Y., Zhong L., Zhang Y., Liu Y., Zheng X., Huang P. (2017). Long Non-Coding RNA NEAT1 Promotes Hepatocellular Carcinoma Cell Proliferation through the Regulation of MiR-129-5p-VCP-IκB. Am. J. Physiol. Gastrointest. Liver Physiol..

[227] Li W., Fu Q., Man W., Guo H., Yang P. (2019). LncRNA OR3A4 Participates in the Angiogenesis of Hepatocellular Carcinoma through Modulating AGGF1/Akt/MTOR Pathway. Eur. J. Pharmacol..

[228] Qi H., Lu Y., Lv J., Wu H., Jing C.L., Zhang C., Zhang S., Bao Q., Zhang X., Xie C. (2018). The Long Noncoding RNA LncPARP1 Contributes to Progression of Hepatocellular Carcinoma through Up-Regulation of PARP1. Biosci. Rep..

[229] Yu A.T., Berasain C., Bhatia S., Rivera K., Liu B., Rigo F., Pappin D.J., Spector D.L. (2021). PHAROH LncRNA Regulates Myc Translation in Hepatocellular Carcinoma via Sequestering TIAR. Elife.

[230] Xiang X., Fu Y., Zhao K., Miao R., Zhang X., Ma X., Liu C., Zhang N., Qu K. (2021). Cellular Senescence in Hepatocellular Carcinoma Induced by a Long Non-Coding RNA-Encoded Peptide PINT87aa by Blocking FOXM1-Mediated PHB2. Theranostics.

[231] Zhang Y., Wen D.-Y., Zhang R., Huang J., Lin P., Ren F.-H., Wang X., He Y., Yang H., Chen G. (2018). A Preliminary Investigation of PVT1 on the Effect and Mechanisms of Hepatocellular Carcinoma: Evidence from Clinical Data, a Meta-Analysis of 840 Cases, and in Vivo Validation. Cell. Physiol. Biochem..

[232] Hongfeng Z., Andong J., Liwen S., Minping B., Xiaowei Y., Mingyong L., Aimin Y. (2020). LncRNA RMRP Knockdown Suppress Hepatocellular Carcinoma Biological Activities via Regulation MiRNA-206/TACR1. J. Cell. Biochem..

[233] Li C., Lu L., Feng B., Zhang K., Han S., Hou D., Chen L., Chu X., Wang R. (2017). The LincRNA-ROR/MiR-145 Axis Promotes Invasion and Metastasis in Hepatocellular Carcinoma via Induction of Epithelial-Mesenchymal Transition by Targeting ZEB2. Sci. Rep..

[234] Zhang Z., Wang S., Yang F., Meng Z., Liu Y. (2020). LncRNA ROR1-AS1 High Expression and Its Prognostic Significance in Liver Cancer. Oncol. Rep..

[235] Jiang X., Wang G., Liu Y., Mei C., Yao Y., Wu X., Chen X., Ma W., Li K., Zhang Z. (2021). A Novel Long Non-Coding RNA RP11-286H15.1 Represses Hepatocellular Carcinoma Progression by Promoting Ubiquitination of PABPC4. Cancer Lett..

[236] Zhu X.-M., Li L., Ren L.-L., Du L., Wang Y.-M. (2021). LncRNA SNHG17 Predicts Poor Prognosis and Promotes Cell Proliferation and Migration in Hepatocellular Carcinoma. Eur. Rev. Med. Pharmacol. Sci..

[237] Liu J., Lu C., Xiao M., Jiang F., Qu L., Ni R. (2017). Long Non-Coding RNA SNHG20 Predicts a Poor Prognosis for HCC and Promotes Cell Invasion by Regulating the Epithelial-to-Mesenchymal Transition. Biomed. Pharmacother..

[238] Shen A., Ma J., Hu X., Cui X. (2020). High Expression of LncRNA-SNHG7 Is Associated with Poor Prognosis in Hepatocellular Carcinoma. Oncol. Lett..

[239] Dong J., Teng F., Guo W., Yang J., Ding G., Fu Z. (2018). LncRNA SNHG8 Promotes the Tumorigenesis and Metastasis by Sponging MiR-149-5p and Predicts Tumor Recurrence in Hepatocellular Carcinoma. Cell. Physiol. Biochem..

[240] Ye S., Ni Y. (2021). LncRNA SNHG9 Promotes Cell Proliferation, Migration, and Invasion in Human Hepatocellular Carcinoma Cells by Increasing GSTP1 Methylation, as Revealed by CRISPR-DCas9. Front. Mol. Biosci..

[241] Hu S., Liu J., Feng S., Wang Y., Liu H. (2021). LncRNA SUMO1P3 Acts as a Prognostic Biomarker and Promotes Hepatocellular Carcinoma Growth and Metastasis. Aging.

[242] Liu W., Huai R., Zhang Y., Rao S., Xiong L., Ding R., Mao C., Zhao W., Hao T., Huang Q. (2019). Down-Regulation Expression of TGFB2-AS1 Inhibits the Proliferation, Migration, Invasion and Induces Apoptosis in HepG2 Cells. Genes Genom..

[243] Chen Y., Huang F., Deng L., Tang Y., Li D., Wang T., Fan Y., Tao Q., Tang D. (2020). Long Non-Coding RNA TGLC15 Advances Hepatocellular Carcinoma by Stabilizing Sox4. J. Clin. Lab. Anal..

[244] Yao J., Hua X., Shi J., Hu X., Lui K., He K., Mai J., Lan T., Lu M. (2022). LncRNA THEMIS2-211, a Tumor-Originated Circulating Exosomal Biomarker, Promotes the Growth and Metastasis of Hepatocellular Carcinoma by Functioning as a Competing Endogenous RNA. FASEB J..

[245] Xu C., Huang Q., Zhang C., Xu W., Xu G., Zhao X., Liu X., Du Y. (2018). Long Non-Coding RNA TRPM2-AS as a Potential Biomarker for Hepatocellular Carcinoma. Ir. J. Med. Sci..

[246] Lu J., Li B., Xiong X., Cheng N. (2020). RNA Sequencing Reveals the Long Noncoding RNA and MRNA Profiles and Identifies Long Non-Coding RNA TSPAN12 as a Potential Microvascular Invasion-Related Biomarker in Hepatocellular Carcinoma. Biomed. Pharmacother..

[247] Yan J., Zhou C., Guo K., Li Q., Wang Z. (2019). A Novel Seven-LncRNA Signature for Prognosis Prediction in Hepatocellular Carcinoma. J. Cell. Biochem..

[248] Gu J.-X., Zhang X., Miao R.-C., Xiang X.-H., Fu Y.-N., Zhang J.-Y., Liu C., Qu K. (2019). Six-Long Non-Coding RNA Signature Predicts Recurrence-Free Survival in Hepatocellular Carcinoma. World J. Gastroenterol..

[249] Zhao X., Bai Z., Li C., Sheng C., Li H. (2020). Identification of a Novel Eight-LncRNA Prognostic Signature for HBV-HCC and Analysis of Their Functions Based on Coexpression and CeRNA Networks. Biomed Res. Int..

[250] Bao L., Zhang M., Han S., Zhan Y., Guo W., Teng F., Liu F., Guo M., Zhang L., Ding G. (2018). MicroRNA-500a Promotes the Progression of Hepatocellular Carcinoma by Post-Transcriptionally Targeting BID. Cell. Physiol. Biochem..

[251] Sadeghpour S., Ghorbian S. (2019). Evaluation of the Potential Clinical Prognostic Value of LncRNA-BANCR Gene in Esophageal Squamous Cell Carcinoma. Mol. Biol. Rep..

[252] Bahramian S., Sahebi R., Roohinejad Z., Delshad E., Javid N., Amini A., Razavi A.E., Shafiee M., Shamsabadi F.T. (2022). Low Expression of LncRNA-CAF Attributed to the High Expression of HIF1A in Esophageal Squamous Cell Carcinoma and Gastric Cancer Patients. Mol. Biol. Rep..

[253] Wu Y., Hu L., Liang Y., Li J., Wang K., Chen X., Meng H., Guan X., Yang K., Bai Y. (2017). Up-Regulation of LncRNA CASC9 Promotes Esophageal Squamous Cell Carcinoma Growth by Negatively Regulating PDCD4 Expression through EZH2. Mol. Cancer.

[254] Liu H., Zhen Q., Fan Y. (2017). LncRNA GHET1 Promotes Esophageal Squamous Cell Carcinoma Cells Proliferation and Invasion via Induction of EMT. Int. J. Biol. Markers.

[255] Li X., Yang H., Wang J., Li X., Fan Z., Zhao J., Liu L., Zang M., Goscinski M.A., Wang J. (2019). High Level of LncRNA H19 Expression Is Associated with Shorter Survival in Esophageal Squamous Cell Cancer Patients. Pathol. Res. Pract..

[256] Liang Y., Wu Y., Chen X., Zhang S., Wang K., Guan X., Yang K., Li J., Bai Y. (2017). A Novel Long Noncoding RNA Linc00460 Up-Regulated by CBP/P300 Promotes Carcinogenesis in Esophageal Squamous Cell Carcinoma. Biosci. Rep..

[257] Zhang S., Liang Y., Wu Y., Chen X., Wang K., Li J., Guan X., Gang X., Yang K., Bai Y. (2019). Upregulation of a Novel LncRNA LINC01980 Promotes Tumor Growth of Esophageal Squamous Cell Carcinoma. Biochem. Biophys. Res. Commun..

[258] Zang B., Zhao J., Chen C. (2019). LncRNA PCAT-1 Promoted ESCC Progression via Regulating ANXA10 Expression by Sponging MiR-508-3p. Cancer Manag. Res..

[259] Dong Z., Liang X., Wu X., Kang X., Guo Y., Shen S., Liang J., Guo W. (2019). Promoter Hypermethylation-Mediated Downregulation of Tumor Suppressor Gene SEMA3B and LncRNA SEMA3B-AS1 Correlates with Progression and Prognosis of Esophageal Squamous Cell Carcinoma. Clin. Exp. Metastasis.

[260] Han G.-H., Lu K.-J., Wang P., Ye J., Ye Y.-Y., Huang J.-X. (2018). LncRNA SNHG16 Predicts Poor Prognosis in ESCC and Promotes Cell Proliferation and Invasion by Regulating Wnt/β-Catenin Signaling Pathway. Eur. Rev. Med. Pharmacol. Sci..

[261] Weng N.-Q., Chi J., Wen J., Mai S.-J., Zhang M.-Y., Huang L., Liu J., Yang X.-Z., Xu G.-L., Fu J.-H. (2020). The Prognostic Value of a Seven-LncRNA Signature in Patients with Esophageal Squamous Cell Carcinoma: A LncRNA Expression Analysis. J. Transl. Med..

[262] Chen J., Shen Z., Deng H., Zhou W., Liao Q., Mu Y. (2018). Long Non-Coding RNA Biomarker for Human Laryngeal Squamous Cell Carcinoma Prognosis. Gene.

[263] Zong M.-Z., Shao Q., An X.-S. (2019). Expression and Prognostic Significance of Long Noncoding RNA AK001796 in Esophageal Squamous Cell Carcinoma. Eur. Rev. Med. Pharmacol. Sci..

[264] Cao T., Shen J., Pan W., Li C., Qiao Z. (2018). Upregulation of Long Noncoding RNA ANRIL Correlates with Tumor Progression and Poor Prognosis in Esophageal Squamous Cell Carcinoma. J. BUON.

[265] Wang M., Li Y., Yang Y., Liu X., Zang M., Li Y., Yang K., Yang W., Zhang S. (2019). Long Non-coding RNA DLX6-AS1 Is Associated with Malignant Progression and Promotes Proliferation and Invasion in Esophageal Squamous Cell Carcinoma. Mol. Med. Rep..

[266] Bao J., Zhou C., Zhang J., Mo J., Ye Q., He J., Diao J. (2018). Upregulation of the Long Noncoding RNA FOXD2-AS1 Predicts Poor Prognosis in Esophageal Squamous Cell Carcinoma. Cancer Biomark..

[267] Zong M.-Z., Feng W.-T., Du N., Yu X.-J., Yu W.-Y. (2019). Upregulation of Long Noncoding RNA LEF1-AS1 Predicts a Poor Prognosis in Patients with Esophageal Squamous Cell Carcinoma. Eur. Rev. Med. Pharmacol. Sci..

[268] Liu D., Gao M., Wu K., Zhu D., Yang Y., Zhao S. (2019). LINC00152 Facilitates Tumorigenesis in Esophageal Squamous Cell Carcinoma via MiR-153-3p/FYN Axis. Biomed. Pharmacother..

[269] Sharma U., Barwal T.S., Khandelwal A., Rana M.K., Rana A.P.S., Singh K., Jain A. (2022). Circulating Long Non-Coding RNAs LINC00324 and LOC100507053 as Potential Liquid Biopsy Markers for Esophageal Squamous Cell Carcinoma: A Pilot Study. Front. Oncol..

[270] Zhang X., Feng Y., Gao Y., Hu J. (2020). Long Noncoding RNA LINC00634 Functions as an Oncogene in Esophageal Squamous Cell Carcinoma Through the MiR-342-3p/Bcl2L1 Axis. Technol Cancer Res Treat.

[271] Yang X.-Z., He Q.-J., Cheng T.-T., Chi J., Lei Z.-Y., Tang Z., Liao Q.-X., Zhang H., Zeng L.-S., Cui S.-Z. (2018). Predictive Value of LINC01133 for Unfavorable Prognosis Was Impacted by Alcohol in Esophageal Squamous Cell Carcinoma. Cell. Physiol. Biochem..

[272] Sun K., Zhao X., Wan J., Yang L., Chu J., Dong S., Yin H., Ming L., He F. (2018). The Diagnostic Value of Long Non-Coding RNA MIR31HG and Its Role in Esophageal Squamous Cell Carcinoma. Life Sci..

[273] Jiao Z., Yu A., Rong W., He X., Zen K., Shi M., Wang T. (2020). Five-LncRNA Signature in Plasma Exosomes Serves as Diagnostic Biomarker for Esophageal Squamous Cell Carcinoma. Aging.

[274] Shi W., Wang Q., Bian Y., Fan Y., Zhou Y., Feng T., Li Z., Cao X. (2019). Long Noncoding RNA PANDA Promotes Esophageal Squamous Carcinoma Cell Progress by Dissociating from NF-YA but Interact with SAFA. Pathol. Res. Pract..

[275] Shen Z., Wu L., Hao W., Li Q., Zhou C. (2020). Expression of the Long Noncoding RNA RP11-169D4.1-001 in Hypopharyngeal Squamous Cell Carcinoma Tissue and Its Clinical Significance. J. Clin. Lab. Anal..

[276] Liang M., Pan Z., Yu F., Chen C. (2020). Long Noncoding RNA SNHG12 Suppresses Esophageal Squamous Cell Carcinoma Progression through Competing Endogenous RNA Networks. Clin. Transl. Oncol..

[277] Zhang Y., Li R., Ding X., Zhang K., Qin W. (2019). Upregulation of Long Non-Coding RNA SNHG6 Promote Esophageal Squamous Cell Carcinoma Cell Malignancy and Its Diagnostic Value. Am. J. Transl. Res..

[278] Wang J., Yang X., Li R., Zhang R., Hu D., Zhang Y., Gao L. (2020). LncRNA SNHG6 Inhibits Apoptosis by Regulating EZH2 Expression via the Sponging of MiR-101-3p in Esophageal Squamous-Cell Carcinoma. Onco Targets.

[279] Qie P., Yin Q., Xun X., Song Y., Zhou S., Liu H., Feng J., Tian Z. (2021). Long Non-Coding RNA SPRY4-IT1 as a Promising Indicator for Three Field Lymph-Node Dissection of Thoracic Esophageal Carcinoma. J. Cardiothorac. Surg..

[280] Wang Y., Zhang W., Liu W., Huang L., Wang Y., Li D., Wang G., Zhao Z., Chi X., Xue Y. (2021). Long Noncoding RNA VESTAR Regulates Lymphangiogenesis and Lymph Node Metastasis of Esophageal Squamous Cell Carcinoma by Enhancing VEGFC MRNA Stability. Cancer Res..

[281] Huang G.-W., Xue Y.-J., Wu Z.-Y., Xu X.-E., Wu J.-Y., Cao H.-H., Zhu Y., He J.-Z., Li C.-Q., Li E.-M. (2018). A Three-LncRNA Signature Predicts Overall Survival and Disease-Free Survival in Patients with Esophageal Squamous Cell Carcinoma. BMC Cancer.

[282] Xie R., Wu S.-N., Gao C.-C., Yang X.-Z., Wang H.-G., Zhang J.-L., Wei Y., Ma T.-H. (2017). Prognostic Value of Combined and Individual Expression of MicroRNA-1290 and Its Target Gene Nuclear Factor I/X in Human Esophageal Squamous Cell Carcinoma. Cancer Biomark..

[283] Zheng S., Zhang X., Wang X., Li J. (2017). Downregulation of MiR-138 Predicts Poor Prognosis in Patients with Esophageal Squamous Cell Carcinoma. Cancer Biomark..

[284] Jin W., Luo W., Fang W., Wang Y., Wang L., Shen Q., Liu W., Zhang H. (2019). MiR-145 Expression Level in Tissue Predicts Prognosis of Patients with Esophageal Squamous Cell Carcinoma. Pathol. Res. Pract..

[285] Gao X., Xie Z., Wang Z., Cheng K., Liang K., Song Z. (2017). Overexpression of MiR-191 Predicts Poor Prognosis and Promotes Proliferation and Invasion in Esophageal Squamous Cell Carcinoma. Yonsei Med. J..

[286] Liu Z., Huang Y., Han Z., Shen Z., Yu S., Wang T., Dong Z., Kang M. (2021). Exosome-Mediated MiR-25/MiR-203 as a Potential Biomarker for Esophageal Squamous Cell Carcinoma: Improving Early Diagnosis and Revealing Malignancy. Transl. Cancer Res.

[287] Yang H., Wei Y.-N., Zhou J., Hao T.-T., Liu X.-L. (2017). MiR-455-3p Acts as a Prognostic Marker and Inhibits the Proliferation and Invasion of Esophageal Squamous Cell Carcinoma by Targeting FAM83F. Eur. Rev. Med. Pharmacol. Sci..

[288] Liu Z.H., Chen L.D., He Y.B., Xu B., Wang K.B., Sun G.X., Zhang Z.H. (2019). Study of Expression Levels and Clinical Significance of MiR-503 and MiR-375 in Patients with Esophageal Squamous Cell Carcinoma. Eur. Rev. Med. Pharmacol. Sci..

[289] Guo Y., Wang C., Miao X., Chen S., Qian Y., Li G., Jiang Y. (2017). Upregulation of Uc.189 in Patients with Esophageal Squamous Cell Carcinoma and Its Clinicopathologic Value. Pathol. Res. Pract..

