# Supplementary material for: Regulatory Roles of Noncoding RNAs in the Progression of Gastrointestinal Cancers and Health Disparities
Source: Cells. 2022 Aug 7;11(15):2448. doi: 10.3390/cells11152448 (PMC9367924; doi:10.3390/cells11152448)
Supplement: Supplementary file 1 [file cells-11-02448-s001.zip › cells-1829485-supplementary.pdf]

Table S1. List of ncRNAs that could be used as therapeutic targets or biomarkers for gastric cancer.

ncRNA	Expression	Clinical significance	Molecular target(s)	Reference
lncRNA AB007962	Down regulated	Prognostic biomarker	unknown	[133]
lncRNA AK025387	Up regulated	Biomarker	MEK and MAPK signaling pathways	[134]
lncRNA CCAL	Up regulated	Biomarker or therapeutic target.	*FOXM1*	[135]
lncRNA CCAT1	Up regulated	Biomarker	unknown	[136]
lncRNA DANCR	Up regulated	Potential biomarker	Cell cycle and metabolism	[137]
lncRNA DDX11-AS1	Up regulated	Biomarker and therapeutic target	MiR-326/*IRS1* Axis	[138]
lncRNA DLX6-AS1	Up regulated	Biomarker and therapeutic target	MiR-204-5p/*OCT1*	[139]
lncRNA EIF3J-DT	UP regulated	Resistance against oxaliplatin and 5FU	*ATG14*	[140]
lncRNA FLJ22763	Down regulated	Prognostic biomarker	*ACLY*	[141]
lncRNA GPC5-AS1	Down regulated	Biomarker and therapeutic target	*GPC5*	[142]
lncRNA H19	Up regulated	Biomarker	unknown	[143]
lncRNA HOTAIR	Up regulated	Prognostic marker	unknown	[144]
lncRNA HOXD-AS2	Down regulated	Biomarker	PI3K/Akt Signaling Pathway	[145]
lncRNA ITPK1-AS1, KCNQ1DN, LINC0016, LINC00173, and LINC00307	Up regulated	Prognostic biomarker	Cell adhesion, cytokine-cytokine receptor interaction, chemokine signaling pathway, leukocyte trans-endothelial migration	[146]
lncRNA KCNQ1OT1	Up regulated	Therapeutic target	miR-145-5p/*ARF6* Axis	[147]
lncRNA linc00152	Up regulated	Biomarker	MicroRNA-193b-3p/ETS1 Axis.	[148]
lncRNA LINC00200	Up regulated	Biomarker and therapeutic target	Hormone secretion	[149]
lncRNA Linc00483	Up regulated	Prognostic biomarker or therapeutic target	MAPKs	[150]
lncRNA LINC00857	Up regulated	Prognostic marker	*Cyclin D1 and E1*	[151]

lncRNA LINC00924	Up regulated	Prognostic biomarker	unknown	[152]
lncRNA LINC00941	Up regulated	Prognostic biomarker	PI3K/AKT Signaling Pathway	[153]
lncRNA LINC01133	Down regulated	Prognostic biomarker and anti-metastatic therapeutic target	Wnt/β-Catenin Pathway	[154]
lncRNA LINC01279	Up regulated	Prognostic biomarker	miR-143-3p/*SERPINE1 Axis*	[155]
lncRNA MALAT1	Up regulated	Therapeutic target	VE-cadherin, β-catenin, MMPs 2 and 9, MT1-MMP, p-ERK, p-FAK, and p-paxillin	[156]
lncRNA MEF2C-AS1 and FENDRR	Up regulated	Biomarkers	*FAT3*, *NTN1* and *LYVE1*	[157]
lncRNA NR_026827	Down regulated	Biomarker in those associated with *H. pylori* infection	unknown	[158]
lncRNA PICART1	Down regulated	Biomarker	PI3K/AKT and MAPK/ERK Signaling Pathways	[159]
lncRNA RP11-397A15.4	Up regulated	Biomarker	unknown	[160]
lncRNA SNHG11	Up regulated	Biomarker	Wnt/β-Catenin Pathway	[161]
lncRNA XLOC_010235	Up regulated	Therapeutic target	*Snail1*	[162]
lncRNA ZEB1-AS1	Up regulated	Prognostic biomarker	*ZEB1*	[163]
lncRNAs hsa_circ_0103398 and hsa_circ_0127859	Up regulated	Prognostic biomarkers	unknown	[164]
lncRNAs HULC and ZNFX1-AS1	Up regulated	Biomarkers	unknown	[165]
miR-122-5p	Down regulated	Therapeutic target	*GIT1*	[166]
miR-125b	Up regulated	Prognostic biomarker	*HER2*	[167]
miR-383	Down regulated	Prognostic biomarker	*SGCZ*	[168]
miRNA-200c, miRNA-139 and lncRNA H19	Up regulated/Down regulated	Biomarkers	*FGF-18*/*FGF-R*	[169]

Table S2. List of ncRNAs that could be used as therapeutic targets or biomarkers for pancreatic cancer.

ncRNA	Expression	Clinical significance	Molecular target(s)	Reference
circRNA ciRS-7	upregulated	Biomarker and therapeutic target	EGFR/STAT3 signaling pathway	[170]
circRNA RTN4	Upregulated	Biomarker	*RAB11FIP1*	[171]
lncRNA H19/miR-675	Upregulated	Diagnostic or prognostic biomarker	*E2F-1*	[172]
lncRNA Linc-ROR	Upregulated	Therapeutic target	*let-7*	[173]
lncRNA AB209630	Downregulated	Inhibits Gemcitabine Resistance	PI3K/AKT Signaling pathway	[174]
lncRNA CERS6-AS1	Upregulated	Therapeutic target	*FGFR1*	[175]
lncRNA DGCR5	Downregulated	Therapeutic target	*PDCD4*	[176]
lncRNA DLEU1	Upregulated	Prognostic biomarker	miR-381/*CXCR4* axis	[177]
lncRNA FEZF1-AS1	Upregulated	Therapeutic target	miR-107/*ZNF312B* axis	[178]
lncRNA H19	Upregulated	Biomarker	PFTK1 and Wnt signaling	[179]
LncRNA HOTTIP	Upregulated	Therapeutic target	*WDR5* and *KRAS*	[180]
lncRNA ITGB2-AS1	Upregulated	Therapeutic target	*RAF1*	[181]
lncRNA LINC00460	Upregulated	Therapeutic target	unknown	[182]
lncRNA LINC00483	Upregulated	Therapeutic target	TBK1/MAPK axis	[183]
lncRNA LINC00657	Upregulated	Therapeutic target	*PAK4*	[184]
lncRNA linc00673/miR-150-5p	Downregulated	Prognostic biomarker	EMT regulator ZEB1	[185]
lncRNA LINC00941	Upregulated	Therapeutic target	Hippo signaling pathway	[186]
lncRNA LINC01234	Up regulated	Therapeutic target	Aspartate metabolism	[187]
lncRNA LUCAT1	Upregulated	Biomarker	miR-539	[188]
lncRNA MALAT1	Upregulated	Diagnostic and prognostic biomarker	*ZEB1, KRAS*	[189,190]
lncRNA MSC-AS1	Upregulated	Therapeutic target	*CDK14*	[191]
lncRNA NEAT1	Upregulated	Biomarker	*RELA*	[192]
lncRNA NONHSAT105177	Downregulated	Therapeutic target	*Clusterin*	[193]

lncRNA OIP5-AS1	Upregulated	Therapeutic target	MiR-429/FOXD1/ERK Pathway	[194]
lncRNA PCED1B-AS1	Upregulated	Biomarker	*HIF-1A*	[195]
lncRNA PTTG3P	Upregulated	Prognostic marker and therapeutic target	*FOXM1*	[196]
lncRNA PVT1	Upregulated	Biomarker	*HIF-1A*	[197]
lncRNA PWAR6	Downregulated	Biomarker and therapeutic target	YAP signaling	[198]
lncRNA THAP9-AS1	Upregulated	Prognostic biomarker	YAP signaling	[199]
lncRNA TP73-AS1	Upregulated	Biomarker and therapeutic target	YAP signaling	[200]

Table S3. List of ncRNAs that could be used as therapeutic targets or biomarkers for liver cancer.

**ncRNA**	**Expression**	**Clinical significance**	**Molecular target(s)**	**Reference**
lncRNA A1BG-AS1	Down regulated	Biomarker	Phosphatase and tensin homolog, and *SMAD7*	[201]
lncRNA AF113014	Down regulated	Therapeutic target	Mir-20a/*Egr2*	[202]
lncRNA AL033381.2	Up regulated	Biomarker and therapeutic target	*PRKRA*	[203]
lncRNA AWPPH	Up regulated	Prognostic biomarker	*YBX1*	[204]
lncRNA CCAT2 and FOXM1	Up regulated	Therapeutic target	miR-34a/ *FOXM1*/ *CCAT2* axis	[205]
lncRNA CDKN2B-AS1	Up regulated	Prognostic biomarker	*CDK2, CDK4 and P16*	[206]
lncRNA D16366	Down regulated	Diagnostic or prognostic biomarker	unknown	[207]
lncRNA FAL1	Up regulated	Biomarker and therapeutic target	miR-1236	[208]
lncRNA GAS5	Down regulated	Therapeutic target	*ANGPTL1*	[209]
lncRNA HOXA11-AS	Up regulated	Therapeutic target	*LATS1*	[210]
lncRNA HOXC13-AS	Up regulated	Prognostic biomarker	unknown	[211]
lncRNA HULC	Up regulated	Therapeutic target	*Sirt1* and *COX-2*	[212,213]
lncRNA LINC00161	Up regulated	Prognostic biomarker	unknown	[214]

lncRNA LINC00324	Up regulated	Therapeutic target	Fas ligand via PU box binding protein	[215]
lncRNA LINC00346	Up regulated	Biomarker	JAK-STAT3 pathway	[216]
lncRNA LINC00963	Up regulated	Biomarker	PI3K/AKT Pathway	[217]
lncRNA LINC01554	Down regulated	Prognostic biomarker	Akt/MTOR Signaling Pathway	[218]
lncRNA lncAY	Up regulated	Biomarker	Myb/MEF2C acetylation	[219]
lncRNA MEG3	Down regulated	Biomarker	*TGF-B1*	[220]
lncRNA MIR4435-2HG	Up regulated	Biomarker and therapeutic target	miRNA-487a, and miR-22-3p/*YWHAZ* axis	[221,222]
lncRNA miR503HG	Down regulated	Prognostic biomarker	HNRNPA2B1/NF-KB signaling pathway	[223]
lncRNA MYLK-AS1	Up regulated	Therapeutic target	EGFR/HER2 and ERK1/2 pathway	[224]
lncRNA NEAT1	Up regulated	Biomarker and therapeutic target	viral carcinogenesis, transcriptional mis regulation in cancer, P53 signaling pathways, miR-129-5p-VCP-IκB	[225,226]
lncRNA OR3A4	Up regulated	Prognostic biomarker	AGGF1/Akt/mTOR pathway	[227]
lncRNA PARP1	Up regulated	Prognostic biomarker	*PARP1*	[228]
lncRNA PHAROH	Up regulated	Biomarker and therapeutic target	*TIAR*	[229]
lncRNA PINT87aa	Up regulated	Therapeutic target	FOXM1/ PHB2 axis	[230]
lncRNA PVT1	Up regulated	Therapeutic target	miR-424-5p/*INCENP* axis	[231]
lncRNA RMRP	Up regulated	Prognostic biomarker	miRNA-206/*TACR1*	[232]
lncRNA RNA-ROR	Up regulated	Therapeutic target	*ZEB2*	[233]
lncRNA ROR1-AS1	Up regulated	Prognostic biomarker	WNT signaling pathway	[234]
lncRNA RP11-286H15.1	Down regulated	Therapeutic target	ubiquitination of PABPC4	[235]
lncRNA SNHG17	Up regulated	Therapeutic target	cell cycle G0/G1 phase arrest and apoptosis	[236]
lncRNA SNHG20	Up regulated	Prognostic biomarker	*ZEB1, ZEB2, N-cadherin, Vimentin, and E-cadherin*	[237]
lncRNA SNHG7	Up regulated	Therapeutic target	*FAIM2*	[238]
lncRNA SNHG8	Up regulated	Prognostic biomarker	miR-149-5p/*PPM1F*	[239]

lncRNA SNHG9	Up regulated	Therapeutic target	GSTP1 Methylation	[240]
lncRNA SUMO1P3	Up regulated	Prognostic marker and therapeutic target	*Cyclin D1* and Akt phosphorylation	[241]
lncRNA TGFB2-AS1	Up regulated	Prognostic biomarker	Apoptosis	[242]
lncRNA TGLC15	Up regulated	Therapeutic target	*SOX4*	[243]
lncRNA THEMIS2-211	Up regulated	Therapeutic target	miR-940/*SPOCK1* axis	[244]
lncRNA TRPM2-AS	Up regulated	Biomarker	*TRPM2*	[245]
lncRNA TSPAN12	Up regulated	Biomarker and therapeutic target	Cell cycle, ECM–receptor interaction, focal adhesion, and p53, PI3K-Akt, and AMPK signaling pathways.	[246]
lncRNAs AC009005.2, RP11-363N22.3, RP11-932O9.10, RP11-572O6.1, RP11-190C22.8, RP11-388C12.8, and ZFPM2-AS1.	Up regulated	Prognostic biomarkers	Cell cycle, spliceosome, Fanconi anemia pathway, DNA replication, mRNA surveillance pathway, homologous recombination, RNA transport, and ubiquitin mediated proteolysis	[247]
lncRNAs MSC-AS1, POLR2J4, EIF3J-AS1, SERHL, RMST, and PVT1.	Up regulated	Prognostic biomarkers	TGF-β signaling and apoptosis pathways	[248]
lncRNAs TSPEAR-AS1, LINC00511, LINC01136, MKLN1-AS, LINC00506, KRTAP5-AS1, ZNF252P-AS1, and THUMPD3-AS1	Up regulated	Prognostic biomarker	Target cell cycle-related biological processes and pathways.	[249]
miR-500a	Up regulated	Prognostic marker and therapeutic target	*BID*	[250]

Table S4. List of ncRNAs that could be used as therapeutic targets or biomarkers for esophageal cancer.

**ncRNA**	**Expression**	**Clinical significance**	**Molecular target(s)**	**Reference**
lncRNA BANCR	Up regulated	Prognostic biomarker	*Notch2*	[251]
lncRNA CAF	Down regulated	Therapeutic target	*HIF-1A*	[252]
lncRNA CASC9	Up regulated	Diagnostic or prognostic biomarker	*PDCD4* expression through EZH2	[253]
lncRNA GHET1	Up regulated	Therapeutic target	*Vimentin, N-cadherin,* and *E-cadherin*	[254]
lncRNA H19	Up regulated	Prognostic biomarker	unknown	[255]
lncRNA Linc00460	Up regulated	Prognostic biomarker	CBP/P300	[256]
lncRNA LINC01980	Up regulated	Prognostic marker and therapeutic target	*GADD45A*	[257]
lncRNA PCAT-1	Up regulated	Therapeutic target	*ANXA10*	[258]
lncRNA SEMA3B and SEMA3B-AS1	Down regulated	Prognostic biomarker	*semaphorin*	[259]
lncRNA SNHG16	Up regulated	Prognostic biomarker	Wnt/β-Catenin Signaling Pathway	[260]
lncRNAs - BQ376030, ASLNC11164, BF894811, RP11-473M20.9, XLOC_007869, XLOC_006476, CK327190	Up regulated	Prognostic biomarkers	Wnt/β-Catenin Signaling Pathway	[261]
lncRNAs AC008440.10	Down regulated	Biomarker	unknown	[262]
lncRNAs AK001796	Up regulated	Prognostic biomarker	SP1/NF-KB pathway	[263]
lncRNAs ANRIL	Up regulated	Prognostic marker and therapeutic target	unknown	[264]
lncRNAs DLX6-AS1	Up regulated	Therapeutic target	*DLX6*	[265]
lncRNAs FOXD2-AS1	Up regulated	Prognostic biomarker	*FOXD2*	[266]
lncRNAs LEF1-AS1	Up regulated	Prognostic biomarker	*LEF1*	[267]

lncRNAs LINC00152	Up regulated	Prognostic biomarker	MiR-153-3p/*FYN* Axis	[268]
lncRNAs LINC00324, LOC100507053	Up regulated and down regulated	Biomarker	hsa-miR-493-5p	[269]
lncRNAs LINC00634	Up regulated	Prognostic biomarker	MiR-342-3p/*Bcl2L1* Axis	[270]
lncRNAs LINC01133	Down regulated	Prognostic biomarker	unknown	[271]
lncRNA MIR31HG	Up regulated	Biomarker	*Furin* and *MMP1*	[272]
lncRNAs NR_039819, NR_036133, NR_003353, ENST00000442416.1, and ENST00000416100.1	Up regulated	Biomarkers	unknown	[273]
lncRNA PANDA	Up regulated	Biomarker and therapeutic target	*NF-YA* and *SAFA*	[274]
lncRNA RP11-169D4.1-001	Down regulated	Biomarker	unknown	[275]
lncRNA SNHG12	Down regulated	Prognostic biomarker	miRNA-195-5p/BCL9	[276]
lncRNA SNHG6	Up regulated	Diagnostic or prognostic biomarker	*EZH2*	[277,278]
lncRNA SPRY4-IT1	Up regulated	Prognostic biomarker	unknown	[279]
lncRNA VESTAR	Up regulated	Therapeutic target	*VEGFC*	[280]
lncRNAs: RP11-366H4.1.1, LINC00460 and AC093850.2	Up regulated	Prognostic biomarker	cell cycle	[281]
miR-1290	Up regulated	Prognostic biomarker	*Nuclear Factor I/X*	[282]
miR-138	Down regulated	Prognostic biomarker	unknown	[283]
miR-145	Down regulated	Prognostic biomarker	*c-Myc*	[284]
miR-191	Up regulated	Therapeutic target	*EGR1*	[285]
miR-25/miR-203	Up regulated and down regulated	Biomarkers	unknown	[286]

miR-455-3p	Down regulated	Prognostic biomarker	*FAM83F*	[287]
miR-503 and miR-375	Up regulated	Biomarker	unknown	[288]
Uc.189	Up regulated	Biomarker	NF-KB signaling pathway	[289]
